# Real-life safety assessment of orally disintegrating desmopressin tablet: Incidence of diagnosed hyponatraemia and other events across three European countries

**DOI:** 10.1016/j.gloepi.2025.100228

**Published:** 2025-10-31

**Authors:** Gunnar Johansson, Jonas Reinold, Nelly L. Shapero, Tekla L. Rosell, Leif A. Jørgensen, Niklas Koenen, Christian Frøsig, Michael Falkenberg, Lene Holdrup, Kristian Juul

**Affiliations:** a Department of Public Health and Caring Sciences, Uppsala University, Uppsala, Sweden; b Department of Clinical Epidemiology, Leibniz Institute for Prevention Research and Epidemiology–BIPS, Bremen, Germany; c Shelbyville, Louisville, KY, USA; d Cambridge University, Cambridge, UK; e Ferring Pharmaceuticals, Södra Hällsjövägen 84, Grängesberg, Sweden; f Department of Biometry and Data Management, Leibniz Institute for Prevention Research and Epidemiology–BIPS, Bremen, Germany; g Ferring Pharmaceuticals, Copenhagen, Denmark

**Keywords:** Desmopressin, Epidemiology, Hyponatraemia, LUTS, Nocdurna, PASS, Safety assessment

## Abstract

**Objective:**

This real-life analysis quantifies the incidence rate of diagnosed hyponatraemia in patients treated with a desmopressin Orally Disintegrating Tablet 25–50 μg (ODT) versus patients treated for lower urinary tract symptoms (LUTS) across Sweden, Denmark, and Germany.

**Material and methods:**

Claims data and register data from drug registries, hospital records, laboratories, and intensive care units were accessed from the three countries in this imposed Post-Authorisation Safety Study (PASS). The incidence rates of diagnosed hyponatraemia, mortality, major cardiovascular events (MACE), venous thromboembolism (VTE), and acute exacerbation of congestive heart failure (CHF) were calculated using Poisson regression. A sensitivity analysis and subgroup analyses of older adult patients and patients with contraindications were conducted. The clinicaltrial.gov number is NCT04740645.

**Results:**

In total, 1,099,545 patients were included in Sweden, Denmark, and Germany. Of these 6745 (0.61 %) were ODT-treated patients, and 1,092,800 (99.39 %) were LUTS-treated patients. The incidence rate of diagnosed hyponatraemia, based on ICD-10 codes, was in the range of 9.04–24.15/1000 patient-years in the ODT cohort and 1.39–4.45/1000 patient-years in the LUTS cohort. Laboratory measured clinically significant hyponatraemia had incidence rates of 139.9 and 33.0/1000 patient-years in the ODT and LUTS cohorts, respectively. The incidence rates of mortality, MACE, and VTE were comparable between groups, with variations in subgroups of patients. Due to different baseline characteristics between the countries, a planned meta-analysis could not be performed.

**Conclusion:**

Results from this real-life study does not indicate any added safety concerns related to use of ODT, since increased rate of hyponatraemia is already in the SmPC.

## Highlights

• We studied over 6000 Nocdurna-treated patients and 1 million reference patients.
• There were increased frequency of low blood sodium in Nocdurna users.
• Mortality and heart failure were not increased for Nocdurna users.
• No added safety concerns for Nocdurna users.

## Introduction

Nocturia, defined by the International Continence Society as waking to pass urine during the main sleep period [1], significantly disrupts sleep and is often linked to underlying medical conditions. Idiopathic nocturnal polyuria, where excess urine is produced at night despite normal overall production, is a common cause. Among 10,190 United States survey respondents with a mean age of 54.4 years, the overall prevalence of nocturnal polyuria was 38.5 % in women and 31.5 % in men, most commonly linked to bladder outlet obstruction in men and to overactive bladder in women [2]. Additionally, approximately 76 % of women and 69 % of men from a randomly selected sample of people aged ≥40 years in the United States, the United Kingdom, and Sweden reported at least 1 nocturnal voiding [3]. Disrupted sleep is associated with daytime tiredness and various health risks, including obesity, diabetes, weakened immune function, and certain cancers [[4], [5], [6], [7], [8]]. The number of voids per night to decrease the quality of life is reported to be at least two [9].

ODT (Nocdurna), a sublingual tablet, targets nocturia attributed to idiopathic nocturnal polyuria. It contains desmopressin, a synthetic analogue of vasopressin, which reduces urine production by promoting water reabsorption in the kidneys. However, some safety concerns have emerged, including the risk of hyponatraemia (especially in older adults aged ≥65 years), thromboembolic events, and exacerbation of congestive heart failure [[10], [11], [12], [13], [14]]. Hyponatraemia, defined as a serum sodium concentration of ≤ 135 mmol/L, is the most common electrolyte disorder in clinical practice, with a prevalence ranging from 15 % to 30 % in hospitalised patients [15]. It is associated with increased morbidity and mortality, particularly in the older adults and those with comorbidities, as it can lead to neurological dysfunction, cognitive impairment, and an increased risk of falls [16,17]. Hyponatraemia has in clinical trials been observed in 14.9 % of ODT-treated patients, while 3.7 % of the placebo group had serum sodium ≤ 135 mmol/L [12,14].

This by the UK imposed post-authorisation safety study (PASS) was conducted to evaluate the safety profile of ODT, including treatment in older patients. The primary objective was to estimate the incidence rate of diagnosed hyponatraemia, defined as a recorded diagnosis of hyponatraemia. The secondary objectives were to calculate the incidence rates of the following; all-cause mortality, major cardiovascular events (MACE), venous thromboembolism (VTE), hyponatraemia requiring intensive care, and low serum sodium. The incidence rate of acute exacerbation of congestive heart failure (CHF) was calculated in a smaller population with pre-existing CHF.

## Materials and methods

This multi-country cohort study utilised data from healthcare sources in three European countries (Sweden, Denmark, and Germany) to identify cohorts of ODT and LUTS patients for analysis of adverse events. The registers used in Denmark and Sweden included records of drug dispensations with Anatomical Therapeutic Chemical (ATC) codes [18,19], causes of death and hospital registers with ICD-10 codes [[20], [21], [22]]. The laboratory database research register with standard laboratory measurements [23] (only in Denmark), and the intensive care register with treatment specifications [24] (only in Sweden) were also included. In Germany, claims data from the German Pharmacoepidemiological Research Database (GePaRD), which includes ICD-10 and ATC-codes and covers approximately 25 million people, was used [26]. In Denmark and Sweden the registers covered the total populations with 5.87 and 10.48 million people, respectively. The study period began 12 months before the launch of ODT (August 2016 or January 2017) in each country and ended on December 31, 2021.

Cohort entry was marked by the index date, defined as the first-ever prescription of ODT during the study period for the ODT cohort [desmopressin; ATC code: H01BA02; brand name: Nocdurna; dose: 25 or 50 microgram (for female and male)], and the first ever prescription of a medication for urinary frequency and incontinence or benign prostatic hyperplasia (ATC codes G04BD and G04C) during the study period for the LUTS cohort (although the ODT cohort could also technically be considered as LUTS patients). Patients were followed from index date until treatment cessation, death, or study end. Patients that qualified for both groups were assigned to the ODT cohort to ensure adverse events caused by ODT were not missed.

Eligibility criteria for the ODT or LUTS cohort included a first-ever ODT or LUTS defining drug prescription, age ≥ 18 at the index date, no previous usage of vasopressin, and ≥ 12 months of registration prior to the index date.

Subpopulations of interest included older adults, patients with a history of CHF, and patients treated despite having contraindications in the ODT cohort. Contraindications for ODT identified from ICD-10 codes used in this study included a history of hyponatraemia, polydipsia, CHF (chronic), chronic kidney disease (stage 3 and above), and syndrome of inappropriate antidiuretic hormone secretion (SIADH).

Descriptive statistics were used to summarise categorical and continuous data. Baseline demographic variables, including gender, ICD-10 codes and ATC codes, were tabulated. Continuous variables such as age, exposure length, hospitalisations, and emergency room visits were included and stratified by country. Incidence rates of the outcomes of interest since the index date were modelled using Poisson regression adjusted for gender and age class, with rates expressed per 1000 patient-years using individual exposure time as the denominator, and with stratum-specific rates analysed by gender and country. No direct comparison between cohorts was planned, since the two groups of patients were expected to be too different at the design phase of the study to qualify for a comparative analysis. The events were defined by the following ICD-10 codes: hyponatraemia (ICD-10 – code E87.1), MACE (ICD-10 - code I21, I22 (myocardial infarction), I63.3-I63.9, I66 (ischemic stroke,)), VTE (ICD-10 - code I26 (pulmonary embolism), I80.1-I80.9, (deep vein thrombosis) and I81 (portal vein thrombosis), and CHF (ICD-10 - code I50 (heart failure)). The events were inpatient events in Denmark and Sweden and both in- and outpatient events in Germany.

A sensitivity analysis where the permissible gap between two subsequent prescriptions was reduced to zero days from the allowed two times the number of prescribed days was conducted. Also, a subgroup analyses of older adult patients and patients with contraindications were conducted.

A comprehensive statistical analysis plan was locked before data was accessed and this plan also specified that if heterogeneity between countries was high no meta-analysis should be done. The study was analysed according to this plan to keep integrity. An amendment to the analysis plan suggested by the authorities was to include an incidence rate calculation of patients on both ODT and LUTS defining drugs and patients on ODT alone. No formal power calculation was made, as this was not a comparative study, but a study imposed by authorities. However, recorded sales data and drug databases screened before the study start estimated the number of possible patients in the ODT cohort to be 7090.

The study had ethical approval in Sweden by Etikprövningsmyndigheten (Dnr 2021–00188), and in Denmark by Sundhedsdatastyrelsen (FSEID-00005459 and FSEID-00005461). In Germany, all involved health insurance providers as well as the German Federal Office for Social Security and the Senator for Health, Women and Consumer Protection in Bremen as their responsible authorities approved the use of German Pharmacoepidemiological Research Database (GePaRD) data for this study. Informed consent for studies based on claims data is required by law unless obtaining consent appears unacceptable and would bias results, which was the case in this study. According to the Ethics Committee of the University of Bremen studies based on GePaRD are exempt from institutional review board review. Data is not available to other researchers from this research team but can be ordered from the three countries. The study was reported to clinicaltrial.gov with the number NCT04740645 and to EU PAS register with the number EUPAS38365.

## Results

The extraction yielded 1,833,893 patients and the analysis included a total of 1,099,545 patients from Sweden, Denmark, and Germany. Among these, 6745 (0.61 %) were in the ODT cohort and 1,092,800 (99.39 %) were in the LUTS cohort. 641 (8.68 %) in the ODT cohort and 733,707 (40.0 %) in the LUTS cohort were excluded from the study. The reasons for exclusion were dispensation of LUTS drugs before study start (93.42 %), less than 12 months of information before study start (3.81 %), age below 18 years (1.84 %), dispensation of vasopressin before study start (0.78 %), missing information about gender (0.09 %) or age (0.05) or two dispensation of vasopressin at study start (0.01 %). Baseline characteristics extracted from medical records or claims data are shown in Table 1.

**Table 1:** Selected baseline characteristics and study duration for included patients during 2016–2021.

	ODT			LUTS		
Variable	Denmark (*n* = 1150)	Germany (*n* = 2701)	Sweden (*n* = 2894)	Denmark (*n* = 151,926)	Germany (*n* = 473,418)	Sweden (*n* = 467,462)
Age, mean (SD)	73.0 (12.8)	72.8 (13.0)	73.1 (12.5)	66.0 (14.8)	64.2 (15.3)	67.3 (15.2)
Males, %	46.2	67.6	33.8	75.2	67.7	75.7
Exposure in days, mean (SD)	155 (243)	196 (275)	199 (199)	395 (455)	293 (377)	651 (621)
Exposure in days, median (IQR)	60 (60, 133)	60 (60, 188)	120 (60, 260)	200 (60, 516)	168 (59, 319)	380 (180, 1128)
Above 65 y, (%)	929 (80.8)	2165 (80.2)	2360 (81.5)	94,254 (62.0)	250,717 (53.0)	309,577 (66.2)
With contraindication, n (%)	26 (2.3)	605 (22.4)	259 (8.9)	N/A	N/A	N/A
Polyuria, ICD-10 = R35, %	4.3	26.1	14.0	0.9	2.9	0.2
Diabetes mellitus, ICD-10 = E10-E14, %	4.2	30.0	4.3	4.5	22.1	2.7
Urinary tract infection, ICD-10 = N39.0, N30, N10, %	6.4	50.8	14.2	6.9	41.6	6.9
Dementia, ICD-10 = F00-F04, G30, G31.0, %	1.1	9.3	12.3	1.0	4.7	8.3
Prostate cancer, ICD-10 = C61, %	3.0	6.9	3.5	4.6	2.0	4.2
Antidiabetics, ATC = A10, %	12.7	18.2	15.7	13.0	14.9	13.1
Diuretics, ATC=C03, %	28.5	40.7	22.3	21.2	28.2	14.5
Overactive bladder, ATC = G04BD, %	32.6	63.9	41.0	34.8	41.0	32.5
Benign prostatic hyperplasia, ATC = G04C, %	25.9	54.2	19.0	65.9	59.7	68.3
Antidepressants, ATC=N06A, %	21.0	43.5	30.1	15.4	32.8	17.1

SD = standard deviation. IQR = Interquartile range.

The baseline characteristics show differences between the countries. The ODT cohort was 5.8–8.6 years older than the LUTS cohort and had more comorbidities and a higher frequency of drug prescriptions (with the exception of benign prostatic hyperplasia medications). Among the eligible patients in the ODT cohort, 66 % were females, and 81.5 % were 65 years or older. The exposure time was longer for the LUTS cohort (293–651 days) than for the ODT cohort (155–199 days).

**Fig. 1:**
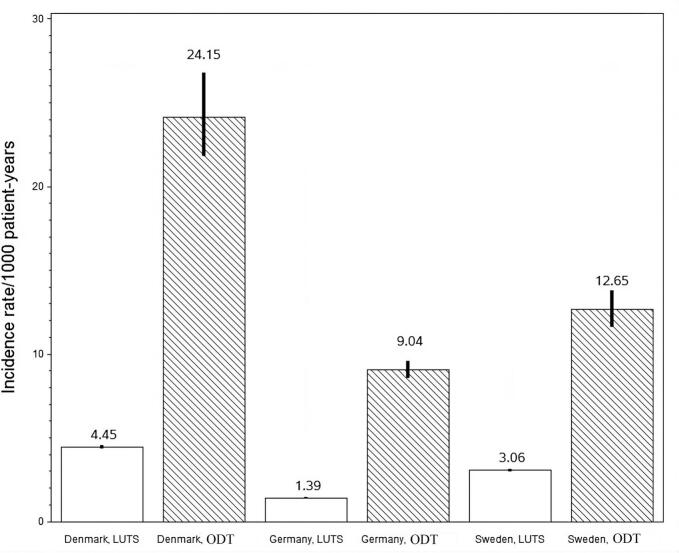
Incidence rates (95 % CI.) of diagnosed hyponatraemia (ICD-10 codes) per 1000 patient-years in Denmark, Sweden and Germany.

**Fig. 2:**
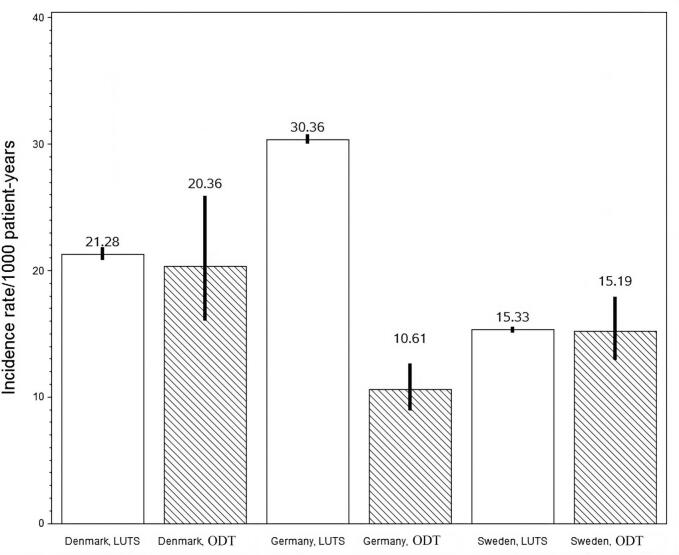
Incidence rates (95 % CI.) of all-cause mortality per 1000 patient-years in Denmark, Sweden, and Germany.

**Fig. 3:**
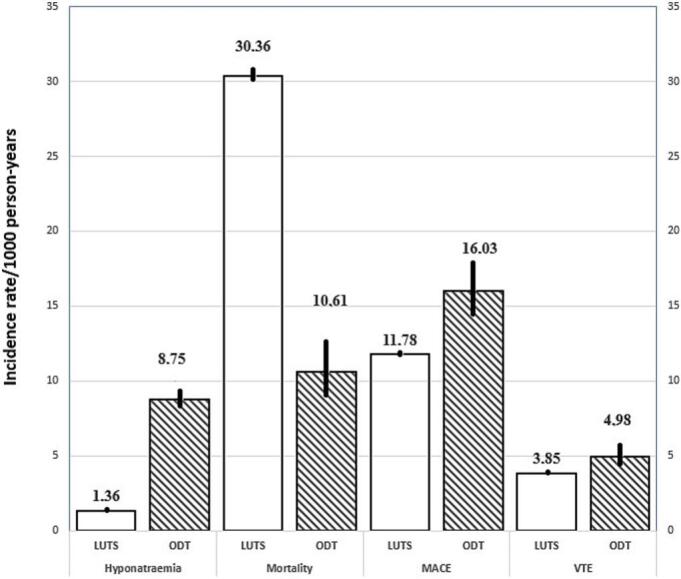
Incidence rates (95 % CI.) per 1000 patient-years of hyponatraemia, mortality, MACE and VTE in Germany.

The results for the primary outcome variable, the incidence rates of diagnosed hyponatraemia, are shown in Fig. 1. Fig. 2 shows the results for one of the secondary outcome variables, the incidence rates of all-cause mortality. Results for both the primary and secondary outcomes are summarised in Table 2 and the results for one country (Germany) is shown in Fig. 3.

**Table 2:** Crude Incidence rates (events/1000 patient-years) of study events with 95 % CI stratified by country during 2016–2021.

Country	Population or time period	Event type	ODT	LUTS
Denmark	All included	Diagnosed hyponatraemia	24.15 (21.75, 26.82)	4.45 (4.36, 4.54)
Germany	All included	Diagnosed hyponatraemia	9.04 (8.54, 9.57)	1.39 (1.37, 1.41)
Sweden	All included	Diagnosed hyponatraemia	12.65 (11.60, 13.79)	3.06 (3.02, 3.10)
Denmark	Age ≥65y	Diagnosed hyponatraemia	36.06 (31.93, 40.72)	6.57 (6.42, 6.72)
Germany	Age ≥65y	Diagnosed hyponatraemia	14.49 (13.50, 15.56)	2.13 (2.09, 2.17)
Sweden	Age ≥65y	Diagnosed hyponatraemia	18.14 (16.39, 20.07)	4.38 (4.32, 4.44)
Sweden	Both drugs^1)^	Diagnosed hyponatraemia	13.18 (10.53, 16.49)	
Denmark	Sensitivity analysis^2)^	Diagnosed hyponatraemia	55.24 (50.32, 60.64)	6.32 (6.19, 6.46)
Sweden	Sensitivity analysis^2)^	Diagnosed hyponatraemia	11.51 (10.82, 12.25)	3.09 (3.04, 3.13)
Denmark	During first month	Diagnosed hyponatraemia	68.36 (63.58, 73.14)	7.51 (7.31, 7.71)
Germany	During first month	Diagnosed hyponatraemia	18.32 (17.38, 19.30)	2.88 (2.83, 2.92)
Sweden	During first month	Diagnosed hyponatraemia	20.21 (18.98, 21.53)	4.28 (4.22, 4.35)
Denmark	After first month	Diagnosed hyponatraemia	9.65 (8.24, 11.30)	3.90 (3.82, 3.99)
Germany	After first month	Diagnosed hyponatraemia	7.15 (6.70, 7.62)	1.18 (1.16, 1.19)
Sweden	After first month	Diagnosed hyponatraemia	9.74 (8.85, 10.72)	2.83 (2.79, 2.87)
Sweden	With contraindication	Diagnosed hyponatraemia	165.6 (142.7, 192.2)	N/A
Germany	With contraindication	Diagnosed hyponatraemia	25.72 ^3)^	N/A
Denmark	All included	All-cause mortality	20.36 (16.01, 25.90)	21.28 (20.78, 21.79)
Germany	All included	All-cause mortality	10.61 (8.91, 12.64)	30.36 (29.99, 30.74)
Sweden	All included	All-cause mortality	15.19 (12.90, 17.88)	15.33 (15.09, 15.57)
Denmark	All included	MACE	4.66 (3.43, 6.33)	5.47 (5.33, 5.61)
Germany	All included	MACE	16.03 (14.34, 17.92)	11.78 (11.62, 11.94)
Sweden	All included	MACE	8.18 (6.76, 9.90)	7.45 (7.34, 7.57)
Denmark	All included	VTE	8.63 (6.55, 11.35)	9.67 (9.48, 9.87)
Germany	All included	VTE	4.98 (4.36, 5.69)	3.85 (3.80, 3.90)
Sweden	All included	VTE	11.42 (9.46, 13.78)	12.23 (12.09, 12.37)

1)This population has 1804 in the ODT cohort also treated with LUTS defining drugs. 2)Sensitivity refers to the sensitivity analysis with a zero day gap allowed between two treatments. 3)The 95 % CI from the Poisson regression cannot be calculated due to low numbers. N/A = Not applicable.

The incidence rate of diagnosed hyponatraemia was in the range of 9.04–24.15/1000 patient-years in the ODT cohort and in the range of 1.39–4.45/1000 patient-years in the LUTS cohort. The lowest rates were observed in Germany, although these were still comparable to the incidence rates in Denmark and Sweden. The analysis of incidence rates during the first month compared to after the first month showed higher event rates during the first month and the increase was of the same magnitude in both cohorts. The incidence rates of all-cause mortality, MACE, and VTE showed variations among countries and treatment groups with no specific pattern. The 95% CI is longer in the ODT cohort than in the LUTS cohort, related to of the lower number of patients. A meta-analysis of outcome variables was not conducted due to heterogeneity in base line characteristics and this heterogeneity was found in all baseline characteristics except for characteristics with a very low incidence rate at baseline.

The subpopulations with a pre-existing CHF were much smaller than the total cohorts, with 40,121, 7585, and 1166 patients in Germany, Sweden, and Denmark, respectively. The incidence rate of CHF exacerbation in the ODT cohort was 40.13 (28.24, 57.04) events/1000 patient-years in Germany and 232.92 (95.14, 570.48) events/1000 patient-years in Sweden. The incidence rate in the LUTS cohort was 39.89 (37.92, 41.97) events/1000 patient-years in Germany and 522.31 (488.91, 558.03) events/1000 patient-years in Sweden. There were too few patients in Denmark to calculate the incidence rates.

The results for laboratory serum sodium measurements, only available in Denmark, are shown in Table 3. A correlation analysis between measured serum sodium and diagnosed hyponatraemia was not planned in the Statistical Analysis Plan and analysis about hospitalization of these patients was not planned.

**Table 3:** Incidence rates (events/1000 patient-years with 95 % CI) of low serum sodium levels and percentage of patients with a recording of low serum sodium in Denmark identified by laboratory measurements during 2017–2021.

Serum Sodium level	ODT (n = 1150)	LUTS (n = 151,926)	% of ODT patients	% of LUTS patients
≤ 125 mmol/L, Severe hyponatraemia	51.97 (46.41, 58.19)	10.15 (9.96, 10.35)	2.97 %	0.69 %
>125 - ≤ 130, Moderate hyponatraemia	108.17 (96.01, 121.87)	29.83 (29.30, 30.37)	4.18 %	1.77 %
>130 - ≤135, Mild hyponatraemia	379.75 (340.14, 423.97)	141.06 (139.00, 143.14)	13.36 %	7.26 %
≤ 130, clinically significant hyponatraemia^⁎)^	139.93 (125.39, 156.16)	32.97 (32.39, 33.55)	6.07 %	1.92 %

Poisson regression used for incidence rates. *) The ≤ 130 mmol/l is a summary of all events below 130 mmol/L. Two measurements had to occur at least 30 days apart to be counted as separate events.

In total, 1,848,459 serum sodium measurements on 153,076 patients were registered in the Danish laboratory database research register, with 1,830,000 measurements in the LUTS cohort and 18,459 measurements in the ODT cohort. These numbers correspond to an average of 16.1 measurements for each patient in the ODT cohort and 12.0 measurements for each patient in the LUTS cohort. 2.96 % of the patients in the ODT cohort and 7.85 % of the patients in the LUTS cohort had no measurements recorded.

The ICU register, available only in Sweden, showed that there were 0 patients in the ODT cohort treated in the ICU following a diagnosis of hyponatraemia, while there were 73 such events in 70 patients in the LUTS cohort, corresponding to an incidence rate of 0.08 (0.08, 0.08) events/1000 patient-years.

Subgroup analysis in the ODT cohort for patients with contraindications to treatment showed higher incidence rates of diagnosed hyponatraemia and mortality compared to those observed in patients without contraindications. In Sweden and Denmark, the patients without contraindications had zero events of diagnosed hyponatraemia. The most common contraindication was pre-existing CHF.

## Discussion

The analysis of baseline characteristics revealed statistically significant differences among the ODT cohorts across the three countries studied, as well as disparities between ODT and LUTS cohorts within each country. This could partly reflect the sampling methods, where for example, a diagnosis of diabetes mellitus is more common in Germany because primary care data is included in the sampling. Consequently, conclusions were drawn based on the incidence rates observed within each country without conducting a meta-analysis. The dissimilarities in baseline characteristics can be attributed to several factors, although specific reasons were not explicitly investigated. The use of national registers in Denmark and Sweden, along with insurance data from Germany, aimed to create comparable study populations. However, variations in prescribing patterns, healthcare systems, and patient demographics likely contributed to the observed differences. For instance, differences in prescription rates could stem from varying medical specialties responsible for ODT prescriptions across countries.

Despite these discrepancies, certain similarities were evident across the studied countries. Notably, the ODT cohort was consistently older than the LUTS cohort, with higher frequencies of comorbidities and medication dispensations/prescriptions at or before the index date. Additionally, the incidence rates of diagnosed hyponatraemia were comparable across countries, albeit higher in the ODT cohort.

The results showed a higher risk of diagnosed hyponatraemia in all ODT patients and especially in patients aged ≥65 years across all countries, which is consistent with findings from multiple previous trials and a strict monitoring of serum sodium is recommended in the Summary of Product Characteristics [10,11,14,27]. Additionally, incidence rates of diagnosed hyponatraemia were consistently higher within the first month of treatment initiation, which again aligns with previous studies [11,14,28,29].

All-cause mortality rates were comparable between ODT and LUTS cohorts in Sweden and Denmark, while the ODT cohort in Germany exhibited a lower mortality rate than the LUTS cohort. This could potentially reflect the fact that ODT is a relatively new drug and therefore less likely to be prescribed to patients with an increased risk of mortality.

The analysis of events of low serum sodium concentration, available only in Denmark, showed higher incidence rates than those observed for diagnosed hyponatraemia from medical records. This can be attributed to the fact that only 1.8 % of all hospitalised patients with low serum sodium get diagnosed with hyponatraemia in Denmark [25]. Compiled data from three clinical trials found that 0.78 % of ODT-treated patients and 0.26 % of patients in the placebo group developed severe hyponatraemia (serum sodium ≤125 mmol/L) [[10], [11], [12]]. These percentages are lower than those found in the laboratory database in this study. The percentage of patients without recorded serum sodium measurements was low and no compensation for this was needed in the statistical analysis. A correlation analysis between measured serum sodium and diagnosed hyponatraemia was not planned in the Statistical Analysis Plan and analysis about hospitalization of the patients was not planned.

The study's limitations include the inherent disparities in data collection methods across countries, such as the availability of ICU information, serum sodium measurements, and primary care data. Additionally, the study design precluded direct comparisons between ODT and LUTS cohorts, necessitating cautious interpretation of results. The Poisson distribution was underdispersed and hence not always fulfilling the assumptions, which also gives some limitation to the results. However, empirical incidence rates reflect the presented patterns. Serum sodium measurements before index date were not available and could have improved understanding of patients at risk of developing hyponatraemia. Nevertheless, the study provides valuable real-life insights into the safety profile of ODT and underscores the importance of considering baseline characteristics and contraindications when prescribing ODT. Also, using epidemiological data from three different gives information about ICU care (Sweden), laboratory measurements (Denmark) and primary care (Germany).

## Conclusion

The results from this real-life study shows an increased incidence rate of hyponatraemia in ODT patients and this does not indicate any added safety concerns related to use of ODT, since an increased rate of hyponatraemia is already highlighted in the summary of product characteristics. The incidence rates of mortality, MACE, and VTE were broadly consistent between groups, with variations in subgroups of patients and countries. The higher incidence rate of mortality in LUTS patients may reflect channeling bias, with healthier patients more likely to receive the newer drug. The incidence rate of VTE in ODT patients were higher than in LUTS patients in Germany. All occurrences of diagnosed hyponatremia were observed in individuals with contraindications in Denmark and Sweden.

## CRediT authorship contribution statement

**Gunnar Johansson:** Writing – review & editing, Methodology, Investigation. **Jonas Reinold:** Writing – review & editing, Validation, Data curation. **Nelly L. Shapero:** Writing – review & editing, Validation. **Tekla L. Rosell:** Writing – original draft, Validation. **Leif A. Jørgensen:** Writing – review & editing, Methodology, Formal analysis, Data curation. **Niklas Koenen:** Writing – review & editing, Formal analysis. **Christian Frøsig:** Writing – review & editing, Resources, Project administration, Funding acquisition. **Michael Falkenberg:** Writing – review & editing. **Lene Holdrup:** Writing – review & editing, Validation. **Kristian Juul:** Writing – review & editing, Supervision, Methodology, Funding acquisition, Conceptualization.

## Consent for publication

Not applicable.

## Ethics approval and consent to participate

The study had ethical approval in Sweden by Etikprövningsmyndigheten (Dnr 2021-00188), and in Denmark by Sundhedsdatastyrelsen (FSEID-00005459 and FSEID-00005461). In Germany, all involved health insurance providers as well as the German Federal Office for Social Security and the Senator for Health, Women and Consumer Protection in Bremen as their responsible authorities approved the use of German Pharmacoepidemiological Research Database (GePaRD) data for this study. Informed consent for studies based on claims data is required by law unless obtaining consent appears unacceptable and would bias results, which was the case in this study. According to the Ethics Committee of the University of Bremen studies based on GePaRD are exempt from institutional review board review.

## Funding

Ferring Pharmaceuticals A/S was the sponsor of this project.

## Declaration of competing interest

Jonas Reinold, Niklas Koenen, Leif Jørgensen, Nelly Shapero, Gunnar Johansson, and Tekla Rosell did consultant work for Ferring Pharmaceuticals. Kristin Juul, Christian Frøsig, Michael Falkenberg and Lene Holdrup are employed by Ferring Pharmaceuticals.

## Data Availability

Data is not available to other researchers from this research team but can be ordered from authorities in the three countries.

## Appendix A Supplementary data

The supplementary tables present data on patient flow, baseline characteristics, and incidence rates of hyponatraemia, ICU admission, mortality, major adverse cardiovascular events (MACE), and venous thromboembolism (VTE), stratified by country and gender. Event rates are also shown for patients with contraindications to ODT treatment and for those with a prior history of congestive heart failure (CHF). Image 1
