# Supplementary material for: Real-life safety assessment of orally disintegrating desmopressin tablet: Incidence of diagnosed hyponatraemia and other events across three European countries
Source: Glob Epidemiol. 2025 Oct 31;10:100228. doi: 10.1016/j.gloepi.2025.100228 (PMC12639583; doi:10.1016/j.gloepi.2025.100228)
Supplement: Supplementary file 1 — The supplementary tables present data on patient flow, baseline characteristics, and incidence rates of hyponatraemia, ICU admission, mortality, major adverse cardiovascular events (MACE), and venous thromboembolism (VTE), stratified by country and gender. Event rates are also shown for patients with contraindications to ODT treatment and for those with a prior history of congestive heart failure (CHF). [file mmc1.docx]

**Patient disposition and flow with number of patients in Sweden, Denmark and Germany.**

|  | **All ODT patients** | **ODT patients, Male** | **ODT patients, Female** | **All LUTS patients** | **LUTS patients, Male** | **LUTS patients, Female** |
| --- | --- | --- | --- | --- | --- | --- |
| **Data from Sweden** | | | | | | |
| Total number of extracted patients | 3,089 | 1,032 | 2,057 | 520,417 | 395,048 | 125,369 |
| **Number of included patients** | **2,894** | **977** | **1,917** | **467,462** | **353,718** | **113,744** |
| Excluded, Below 18 y | 1 | 0 | 1 | 445 | 156 | 289 |
| Excluded, >= 1 dispensation of ODT | 11 | 5 | 6 | 0 | 0 | 0 |
| Excluded, vasopressin treated | 184 | 50 | 134 | 1,828 | 870 | 958 |
| Excluded, <12 month records before index date | 0 | 0 | 0 | 98 | 72 | 26 |
| LUTS drugs before study period start | 0 | 0 | 0 | 50,752 | 40,319 | 10,433 |
| *ODT and LUTS treated’*, assigned to ODT, not excluded | 1,922 | 777 | 1,145 | N/A | N/A | N/A |
| Sum excluded | 195 | 55 | 140 | 52,955 | 41,330 | 11,625 |
| Number of patients in the CHF cohort, | 49 | 21 | 28 | 7,536 | 6,176 | 1,360 |
| **Data from Denmark** | | | | | | |
|  | **All ODT patients** | **ODT patients, Male** | **ODT patients, Female** | **All LUTS patients** | **LUTS patients, Male** | **LUTS patients, Female** |
| Total number of extracted patients | 1,455 | 669 | 786 | 274,020 | 207,424 | 66,596 |
| **Number of included patients** | **1,150** | **531** | **619** | **151,926** | **114,229** | **37,697** |
| Excluded, Below 18 y | 8 | Diskr | Diskr | 5,008 | 3,080 | 1,928 |
| Excluded, >= 1 dispensation of ODT | 0 | 0 | 0 | 0 | 0 | 0 |
| Excluded, vasopressin treated | 301 | 135 | 166 | 1,247 | 779 | 468 |
| LUTS drugs before study period start | 0 | 0 | 0 | 116,362 | 89,379 | 26,983 |
| Not in population 1 year before | Diskr | Diskr | Diskr | 2,134 | 1,805 | 329 |
| *‘ODT and LUTS treated’*, assigned to ODT, not excluded | 915 | 522 | 393 | N/A | N/A | N/A |
| Sum excluded | 305 | 138 | 167 | 122,094 | 93,195 | 28,899 |
| Number of patients in the CHF cohort, | 16 | 9 | 7 | N/A | N/A | N/A |
| **Data from Germany** | | | | | | |
|  | **All ODT patients** | **ODT patients, Male** | **ODT patients, Female** | **All LUTS patients** | **LUTS patients, Male** | **LUTS patients, Female** |
| Number of extracted patients | 2,842 | 1,925 | 917 | 1,032,648 | 746,452 | 286,185 |
| Excluded, dispensation of respective study drug any time prior | 0 | 0 | 0 | 523,855 | 401,855 | 121,999 |
| Excluded, missing or inconsistent information on age | 0 | 0 | 0 | 347 | 234 | 108 |
| Excluded, missing or inconsistent information on sex or “diverse” sex | 3 | N/A | N/A | 683 | N/A | N/A |
| Excluded, no valid information on residency in Germany | 6 | 5 | 1 | 2,596 | 1,985 | 603 |
| Excluded, age < 18y | 23 | 14 | 9 | 16,323 | 9,754 | 6,564 |
| Excluded, <12 months records before cohort entry | 32 | 29 | 3 | 23,698 | 17,865 | 5,825 |
| Excluded, Vasopressin treated 180 days prior cohort entry | 87 | 54 | 33 | 2,39 | 1,542 | 697 |
| Excluded, Desmopressin (other than ODT) treated on cohort entry | 0 | 0 | 0 | N/A | N/A | N/A |
| Excluded, sum of excluded patients | 141 | 98 | 43 | 558,658 | 425,697 | 132,950 |
| In both groups, assigned to ODT group | 572 | 371 | 201 | N/A | N/A | N/A |
| Final study population | 2,701 | 1,827 | 874 | 473,418 | 320,384 | 153,034 |
| Number of patients in the HF cohort | 390 | 273 | 117 | 39,731 | 27,224 | 12,507 |

Diskr: The numbers in the cell is too low to be shown due to GDPR rules.

**Baseline characteristics for categorical variables, gender, drug usage, and disease codes, with heterogeneity and p-values for test for homogeneity analysing the ODT and LUTS patients within each country.**

|  | **ODT** | | | **LUTS** | | |  |  |
| --- | --- | --- | --- | --- | --- | --- | --- | --- |
| **Variable** | **Denmark (n=1,150)** | **Germany (n=2,701)** | **Sweden (n=2,894)** | **Denmark (n= 151,926)** | **Germany (n= 473,418)** | **Sweden (n= 467,462)** | **Hetero-geneity** | **p value** |
| ACE inhibitors, % | 13.9 | 50.5 | 18.1 | 15.4 | 39.2 | 15.9 | 95.99 | <.0001 |
| Amiodarone, % | 0.4 | 2.7 | 0.1 | 0.7 | 1.6 | 0.3 | 79.20 | 0.0082 |
| Angiotensin II receptor blockers (combinations), % | 8.7 | 23.2 | 7.7 | 6.8 | 14.9 | 6.0 | 85.93 | 0.0008 |
| Angiotensin II receptor blockers, % | 16.9 | 33.3 | 23.4 | 15.8 | 22.9 | 16.4 | 92.06 | <.0001 |
| Anti-inflammatory agents - non-steroids, 'M01A', % | 33.4 | 93.4 | 41.3 | 31.9 | 87.4 | 27.3 | 96.64 | <.0001 |
| Antidepressants, 'N06A', % | 21.0 | 43.5 | 30.1 | 15.4 | 32.8 | 17.1 | 89.36 | <.0001 |
| Antidiabetics, 'A10', % | 12.7 | 18.2 | 15.7 | 13.0 | 14.9 | 13.1 | 71.55 | 0.0298 |
| Antiepileptic, 'N03A', % | 10.7 | 25.7 | 11.7 | 9.4 | 15.1 | 6.1 | 93.21 | <.0001 |
| Antihypertensive, 'C02', % | 1.9 | 11.5 | 2.1 | 1.8 | 6.0 | 1.7 | 89.11 | 0.0001 |
| Atrial fibrillation, 'I48', % | 5.6 | 17.5 | 4.3 | 4.8 | 11.4 | 3.9 | 88.46 | 0.0002 |
| Benign prostatic hyperplasia, 'G04C', % | 25.9 | 54.2 | 19.0 | 65.9 | 59.7 | 68.3 | 99.84 | <.0001 |
| Benign prostatic hyperplasia, N40, D291, ATC=G0, % | 26.3 | 50.8 | 20.0 | 66.1 | 38.6 | 69.0 | 99.92 | <.0001 |
| Beta blocking agents, 'C07', % | 20.8 | 53.4 | 0.0 | 20.0 | 41.8 | 0.0 | 96.21 | <.0001 |
| Bladder cancer, 'C67', % | 0.3 | 2.7 | 0.9 | 0.5 | 1.6 | 1.1 | 83.84 | 0.0021 |
| Calcium channel blockers and diuretics, 'C08G', % | 0.0 | 0.1 | 0.0 | 0.0 | 0.0 | 0.0 | N/A | N/A |
| Cancer, C00-C43, C45-C97, % | 7.0 | 19.0 | 10.1 | 8.6 | 11.2 | 8.8 | 96.94 | <.0001 |
| Carbamazepine and lamotrigine, 'N03AF01' 'N03AX0, % | 2.2 | 4.3 | 1.8 | 1.6 | 2.4 | 1.3 | 25.78 | 0.2599 |
| Chlorpromazine, 'N05AA01', % | 0.0 | 0.0 | 0.0 | 0.0 | 0.0 | 0.0 | N/A | N/A |
| Chronic lung disease, J40-J47, J60-J70, J80, J82, % | 4.7 | 35.5 | 3.2 | 4.3 | 28.9 | 2.1 | 49.79 | 0.1365 |
| Cystic fibrosis, 'E84', % | 0.0 | 0.0 | 0.0 | 0.0 | 0.0 | 0.0 | N/A | N/A |
| Dementia F00, F01, F02, F03, F04, G30, G31.0, % | 1.1 | 9.3 | 12.3 | 1.0 | 4.7 | 8.3 | 84.22 | 0.0018 |
| Desmopressin, 'H01BA02', % | 100.0 | 100.0 | 100.0 | 0.2 | 0.1 | 0.1 | N/A | N/A |
| Diabetes insipidus, 'E232', % | 0.0 | 0.6 | 0.2 | 0.0 | 0.1 | 0.0 | 90.55 | <.0001 |
| Diabetes mellitus, 'E10' 'E11' 'E12' 'E13' 'E14', % | 4.2 | 30.0 | 4.3 | 4.5 | 22.1 | 2.7 | 82.68 | 0.0031 |
| Diuretics, 'C03', % | 28.5 | 40.7 | 22.3 | 21.2 | 28.2 | 14.5 | 56.28 | 0.1015 |
| Gender/Males, % | 46.2 | 67.6 | 33.8 | 75.2 | 67.7 | 75.7 | 99.92 | <.0001 |
| Heart failure, 'I50', % | 1.2 | 14.7 | 0.9 | 1.8 | 9.2 | 1.8 | 95.75 | <.0001 |
| Hyperlipidaemi, 'E78', % | 2.3 | 47.7 | 0.2 | 2.4 | 33.6 | 0.1 | 78.47 | 0.0096 |
| Hypertension, 'I10', % | 10.2 | 70.4 | 3.8 | 8.3 | 54.1 | 1.9 | 90.44 | <.0001 |
| Kidney disease, 'N18' 'N19', % | 0.4 | 18.2 | 0.7 | 1.5 | 10.8 | 1.0 | 94.93 | <.0001 |
| Lipid-modifying agents, 'C10', % | 39.2 | 48.8 | 35.9 | 35.2 | 35.1 | 29.2 | 94.84 | <.0001 |
| Lithium, 'N05AN01', % | 0.7 | 1.0 | 0.9 | 0.3 | 0.7 | 0.4 | 0.00 | 0.9088 |
| Liver disease, K70-K77, F10, ATC=N07BB, % | 12.3 | 22.8 | 27.7 | 13.0 | 17.9 | 17.9 | 95.74 | <.0001 |
| Loperamide, 'A07DA03', % | 2.1 | 6.9 | 5.3 | 1.1 | 5.1 | 2.4 | 90.20 | <.0001 |
| NSAIDs, 'M01A', % | 33.4 | 93.4 | 41.3 | 31.9 | 87.4 | 27.3 | 97.04 | <.0001 |
| Non-selective monoamine reuptake inhibitors, 'N0’, % | 3.3 | 30.3 | 7.0 | 2.6 | 21.4 | 3.4 | 86.52 | 0.0006 |
| Nykturi, 'R359B', % | 1.2 | N/A | 12.4 | 0.6 | N/A | 0.1 | 99.83 | <.0001 |
| Obesity, 'E66', % | 1.6 | 36.0 | 0.4 | 1.7 | 31.3 | 0.4 | 0.00 | 0.4792 |
| Opioids, 'N02A', % | 31.4 | 47.7 | 38.2 | 22.3 | 35.6 | 21.7 | 94.60 | <.0001 |
| Overactive bladder, 'G04BD', % | 32.6 | 63.9 | 41.0 | 34.8 | 41.0 | 32.5 | 99.10 | <.0001 |
| Overactive bladder, N310, N312, N319, N394, R391, % | 47.1 | 16.0 | 50.6 | 46.3 | 5.1 | 35.1 | 99.22 | <.0001 |
| Phenothiazines with aliphatic side-chain, 'N05AA, % | 0.3 | 0.4 | 0.2 | 0.2 | 0.3 | 0.2 | 0.00 | 0.4855 |
| Pollakisuri, 'R359C', % | 0.0 | N/A | 0.2 | 0.0 | N/A | 0.0 | N/A | N/A |
| Polydipsia, 'R361', % | 0.0 | 0.3 | 0.0 | 0.0 | 0.1 | 0.0 | N/A | N/A |
| Polyuria with an A, 'R359A', % | 2.9 | N/A | 0.5 | 0.0 | N/A | 0.0 | 87.07 | 0.0054 |
| Polyuria, 'R35', % | 4.3 | 26.1 | 14.0 | 0.9 | 2.9 | 0.2 | 99.78 | <.0001 |
| Pre-eclampsia, 'O11' 'O14', % | 0.0 | 0.0 | 0.0 | 0.0 | 0.1 | 0.0 | 19.90 | 0.2870 |
| Prostate cancer, 'C61', % | 3.0 | 6.9 | 3.5 | 4.6 | 2.0 | 4.2 | 99.04 | <.0001 |
| Selective serotonin reuptake inhibitors, 'N06AB', % | 11.2 | 18.2 | 15.8 | 8.3 | 14.8 | 9.6 | 90.42 | <.0001 |
| Special ACE inhibitors, 'C09BA', % | 3.7 | 20.8 | 2.2 | 3.9 | 14.2 | 2.5 | 93.09 | <.0001 |
| Special beta blocking agents, 'C07B' 'C07C' 'C07, % | 0.3 | 5.9 | 0.0 | 0.1 | 3.7 | 0.0 | 52.72 | 0.1459 |
| Special diuretics, 'C03A' 'C03B' 'C03C' 'C03D', % | 28.5 | 40.7 | 22.3 | 21.2 | 28.2 | 14.5 | 55.67 | 0.1048 |
| Sulfonylureas including chlorpropamide, 'A10BB', % | 1.0 | 5.7 | 0.0 | 1.4 | 3.8 | 0.0 | 82.67 | 0.0163 |
| Systemic corticosteroids, 'H02', % | 12.7 | 51.9 | 21.0 | 9.0 | 41.7 | 11.3 | 93.62 | <.0001 |
| Urinary tract infection, N39.0, N30, N10, % | 6.4 | 50.8 | 14.2 | 6.9 | 41.6 | 6.9 | 97.02 | <.0001 |
| Vasopressin and analogues (incl. desmopressin), % | 100.0 | 100.0 | 100.0 | 0.2 | 0.1 | 0.1 | . | N/A |

**Incidence rate of diagnosed hyponatraemia, ICU visits, mortality, major cardiovascular and major venous thromboembolic events including sensitivity analysis in Sweden, Denmark, and Germany.**

|  | **All ODT patients** | **ODT, Male** | **ODT, Female** | **All LUTS patients** | **LUTS, Male** | **LUTS, Female** |
| --- | --- | --- | --- | --- | --- | --- |
| **Incidence rate of diagnosed hyponatraemia in Sweden** | | | | | | |
| **All patients – All exposure time** |  |  |  |  |  |  |
| Number of subjects | 2,894 | 977 | 1,917 | 467,462 | 353,718 | 113,744 |
| Mean unadjusted rate of events (events/1000 person-years) | 19.62 | 18.20 | 20.37 | 3.22 | 2.94 | 4.56 |
| Incidence rate (95 % CI) | 12.65 (11.60, 13.79) | 14.65 (12.58, 17.06) | 12.99 (11.70, 14.42) | 3.06 (3.02, 3.10) | 2.40 (2.37, 2.44) | 3.80 (3.72, 3.89) |
| **First month after Index date** |  |  |  |  |  |  |
| Number of subjects | 2,894 | 977 | 1,917 | 467,462 | 353,718 | 113,744 |
| Mean unadjusted rate of events (events/1000 person-years) | 29.49 | 37.45 | 25.44 | 4.72 | 4.54 | 5.25 |
| Incidence rate (95 % CI) | 20.21 (18.98, 21.53) | 29.29 (26.68, 32.16) | 15.38 (14.06, 16.81) | 4.28 (4.22, 4.35) | 3.95 (3.89, 4.02) | 4.47 (4.34, 4.60) |
| **After first month and onwards** |  |  |  |  |  |  |
| Number of subjects | 2,840 | 960 | 1,880 | 460,521 | 348,474 | 112,047 |
| Mean unadjusted rate of events (events/1000 person-years) | 15.22 | 12.77 | 16.53 | 3.01 | 2.75 | 4.22 |
| Incidence rate (95 % CI) | 9.74 (8.85, 10.72) | 10.18 (8.53, 12.14) | 10.53 (9.42, 11.78) | 2.83 (2.79, 2.87) | 2.23 (2.19, 2.26) | 3.52 (3.44, 3.59) |
| **Patients >= 65 years** |  |  |  |  |  |  |
| Number of subjects | 2,360 | 777 | 1,583 | 309,577 | 245,412 | 64,165 |
| Mean unadjusted rate of events (events/1000 person-years) | 23.24 | 20.71 | 24.52 | 3.77 | 3.35 | 6.29 |
| Incidence rate (95 % CI) | 18.14 (16.39, 20.07) | 19.47 (16.23, 23.34) | 21.13 (18.63, 23.97) | 4.38 (4.32, 4.44) | 3.21 (3.17, 3.26) | 5.90 (5.75, 6.05) |
| **Incidence rate of diagnosed hyponatraemia in Denmark** | | | | | | |
| **All patients - All exposure time** |  |  |  |  |  |  |
| Number of subjects | 1,150 | 531 | 619 | 151,926 | 114,229 | 37,697 |
| Mean unadjusted rate of events (events/1000 person-years) | 36.81 | 31.79 | 40.92 | 4.42 | 3.88 | 6.95 |
| Incidence rate (95 % CI) | 24.15 (21.75, 26.82) | 24.91 (21.15, 29.34) | 21.88 (18.96, 25.25) | 4.45 (4.36, 4.54) | 3.46 (3.39, 3.54) | 4.99 (4.77, 5.21) |
| **First month after Index date** |  |  |  |  |  |  |
| Number of subjects | 1,150 | 531 | 619 | 151,926 | 114,229 | 37,697 |
| Mean unadjusted rate of events (events/1000 person-years) | 116.46 | 138.12 | 98.74 | 8.04 | 7.48 | 9.71 |
| Incidence rate (95 % CI) | 68.36 (63.58, 73.14) | 100.34 (91.45, 109.23) | 38.62 (33.28, 43.96) | 7.51 (7.31, 7.71) | 6.47 (6.25, 6.69) | 5.33 (4.91, 5.75) |
| **After first month and onwards** |  |  |  |  |  |  |
| Number of subjects | 1,054 | 442 | 612 | 143,722 | 109,835 | 33,887 |
| Mean unadjusted rate of events (events/1000 person-years) | 14.55 | 4.69 | 22.39 | 3.83 | 3.36 | 5.98 |
| Incidence rate (95 % CI) | 9.65 (8.24, 11.30) | 3.73 (2.48, 5.61) | 12.27 (10.22, 14.74) | 3.90 (3.82, 3.99) | 3.02 (2.96, 3.09) | 4.38 (4.19, 4.58) |
| **Patients >= 65 years** |  |  |  |  |  |  |
| Number of subjects | 929 | 437 | 492 | 94,254 | 73,206 | 21,048 |
| Mean unadjusted rate of events (events/1000 person-years) | 43.72 | 36.97 | 49.47 | 5.53 | 4.64 | 10.31 |
| Incidence rate (95 % CI) | 36.06 (31.93, 40.72) | 35.36 (29.34, 42.60) | 39.29 (32.94, 46.87) | 6.57 (6.42, 6.72) | 4.60 (4.50, 4.71) | 8.67 (8.24, 9.11) |
| **Incidence rate of diagnosed hyponatraemia in Germany** | | | | | | |
| **All patients - All exposure time** |  |  |  |  |  |  |
| Number of subjects | 2,701 | 1,827 | 874 | 473,418 | 320,384 | 153,034 |
| Mean unadjusted rate of events (events/1000 person-years) | 14.63 | 12.03 | 19.19 | 1.41 | 1.08 | 2.89 |
| Incidence rate (95 % CI) | 9.04 (8.54, 9.57) | 7.33 (6.78, 7.93) | 10.45 (9.58, 11.40) | 1.39 (1.37, 1.41) | 0.91 (0.90, 0.93) | 1.95 (1.90, 2.00) |
| **First month after Index date** |  |  |  |  |  |  |
| Number of subjects | 2,701 | 1,827 | 874 | 473,418 | 320,384 | 153,034 |
| Mean unadjusted rate of events (events/1000 person-years) | 31.69 | 20.08 | 55.95 | 3.22 | 2.28 | 5.32 |
| Incidence rate (95 % CI) | 18.32 (17.38, 19.30) | 10.86 (10.13, 11.64) | 29.98 (27.53, 32.65) | 2.88 (2.83, 2.92) | 1.95 (1.91, 1.98) | 4.21 (4.10, 4.33) |
| **After first month and onwards** |  |  |  |  |  |  |
| Number of subjects | 2,411 | 1,551 | 860 | 425,794 | 305,124 | 120,670 |
| Mean unadjusted rate of events (events/1000 person-years) | 11.53 | 10.45 | 13.35 | 1.21 | 0.97 | 2.41 |
| Incidence rate (95 % CI) | 7.15 (6.70, 7.62) | 6.31 (5.79, 6.87) | 7.07 (6.39, 7.82) | 1.18 (1.16, 1.19) | 0.81 (0.80, 0.83) | 1.50 (1.46, 1.55) |
| **Patients ≥ 65 years** |  |  |  |  |  |  |
| Number of subjects | 2,165 | 1,495 | 670 | 250,717 | 172,675 | 78,042 |
| Mean unadjusted rate of events (events/1000 person-years) | 18.14 | 14.33 | 25.66 | 1.95 | 1.41 | 4.57 |
| Incidence rate (95 % CI) | 14.49 (13.50, 15.56) | 10.77 (9.80, 11.84) | 19.58 (17.50, 21.91) | 2.13 (2.09, 2.17) | 1.26 (1.24, 1.29) | 3.49 (3.37, 3.61) |
| **Incidence of diagnosed hyponatraemia requiring care at an Intensive Care Unit (ICU), Sweden** | | | | | | |
| All patients – All exposure time |  |  |  |  |  |  |
| Number of subjects | 2,894 | 977 | 1,917 | 467,462 | 353,718 | 113,744 |
| Mean unadjusted rate of events (events/1000 person-years) | 0.00 | 0.00 | 0.00 | 0.09 | 0.09 | 0.10 |
| Incidence rate (95 % CI) | 2.89E-10 (2.84E-10, 2.94E-10) | 8.62E-10 (8.48E-10, 8.76E-10) | 8.7E-11 (8.41E-11, 8.99E-11) | 0.08 (0.08, 0.08) | 0.08 (0.07, 0.08) | 0.07 (0.07, 0.07) |
| **First month after Index date** |  |  |  |  |  |  |
| Number of subjects | 2,894 | 977 | 1,917 | 467,462 | 353,718 | 113,744 |
| Mean unadjusted rate of events (events/1000 person-years) | 0.00 | 0.00 | 0.00 | 0.08 | 0.10 | 0.00 |
| Incidence rate (95 % CI) | 4.39E-20 (0, Infinity) | 2.4E-14 (255E-265, 2.25E235) | 1.17E-18 (0, Infinity) | 3.01E-10 (0, Infinity) | 0.000085 (901E-256, 7.95E244) | 1.17E-18 (0, Infinity) |
| **After first month and onwards** |  |  |  |  |  |  |
| Number of subjects | 2,840 | 960 | 1,880 | 460,521 | 348,474 | 112,047 |
| Mean unadjusted rate of events (events/1000 person-years) | 0.00 | 0.00 | 0.00 | 0.08 | 0.08 | 0.10 |
| Incidence rate (95 % CI) | 2.69E-10 (2.65E-10, 2.74E-10) | 8.16E-10 (8.02E-10, 8.3E-10) | 8.35E-11 (8.08E-11, 8.63E-11) | 0.08 (0.08, 0.08) | 0.07 (0.07, 0.07) | 0.07 (0.07, 0.07) |
| **Patients >= 65 years** |  |  |  |  |  |  |
| Number of subjects | 2,360 | 777 | 1,583 | 309,577 | 245,412 | 64,165 |
| Mean unadjusted rate of events (events/1000 person-years) | 0.00 | 0.00 | 0.00 | 0.10 | 0.09 | 0.14 |
| Incidence rate (95 % CI) | 8.78E-10 (8.62E-10, 8.94E-10) | 2.407E-9 (2.368E-9, 2.448E-9) | 1.38E-10 (1.34E-10, 1.43E-10) | 0.12 (0.11, 0.12) | 0.09 (0.09, 0.09) | 0.14 (0.13, 0.14) |
| **Sensitivity analysis, Incidence of diagnosed hyponatraemia in Sweden with second prescription shifted to the end of the first** | | | | | | |
| **All patients – All exposure time** |  |  |  |  |  |  |
| Number of subjects | 2,894 | 977 | 1,917 | 467,462 | 353,718 | 113,744 |
| Mean unadjusted rate of events (events/1000 person-years) | 16.78 | 18.91 | 15.69 | 3.30 | 3.16 | 3.93 |
| Incidence rate (95 % CI) | 11.51 (10.82, 12.25) | 15.32 (13.85, 16.94) | 10.18 (9.43, 11.00) | 3.09 (3.04, 3.13) | 2.66 (2.62, 2.70) | 3.56 (3.48, 3.65) |
| **First month after Index date** |  |  |  |  |  |  |
| Number of subjects | 2,894 | 977 | 1,917 | 467,462 | 353,718 | 113,744 |
| Mean unadjusted rate of events (events/1000 person-years) | 29.49 | 37.45 | 25.44 | 4.64 | 4.48 | 5.14 |
| Incidence rate (95 % CI) | 20.35 (19.11, 21.67) | 29.37 (26.77, 32.24) | 15.60 (14.28, 17.05) | 4.22 (4.16, 4.29) | 3.90 (3.84, 3.97) | 4.42 (4.30, 4.55) |
| **After first month and onwards** |  |  |  |  |  |  |
| Number of subjects | 1,581 | 556 | 1,025 | 378,202 | 300,462 | 77,740 |
| Mean unadjusted rate of events (events/1000 person-years) | 12.22 | 12.85 | 11.90 | 2.58 | 2.50 | 2.95 |
| Incidence rate (95 % CI) | 8.44 (7.84, 9.08) | 10.27 (9.10, 11.60) | 8.01 (7.30, 8.79) | 2.37 (2.33, 2.41) | 2.07 (2.04, 2.10) | 2.72 (2.64, 2.79) |
| **Patients >= 65 years** |  |  |  |  |  |  |
| Number of subjects | 2,360 | 777 | 1,583 | 309,577 | 245,412 | 64,165 |
| Mean unadjusted rate of events (events/1000 person-years) | 19.18 | 20.56 | 18.52 | 3.92 | 3.63 | 5.70 |
| Incidence rate (95 % CI) | 15.77 (14.68, 16.95) | 19.31 (17.12, 21.78) | 16.29 (14.88, 17.83) | 4.33 (4.26, 4.40) | 3.48 (3.43, 3.53) | 5.43 (5.27, 5.59) |
| **Sensitivity analysis. Incidence of diagnosed hyponatraemia in Denmark with second prescription shifted to the end of the first.** | | | | | | |
| All patients - All exposure time |  |  |  |  |  |  |
| Number of subjects | 1,150 | 531 | 619 | 151,926 | 114,229 | 37,697 |
| Mean unadjusted rate of events (events/1000 person-years) | 84.83 | 101.63 | 72.80 | 5.66 | 4.89 | 10.45 |
| Incidence rate (95 % CI) | 55.24 (50.32, 60.64) | 80.87 (70.97, 92.14) | 43.53 (38.09, 49.75) | 6.32 (6.19, 6.46) | 4.46 (4.37, 4.56) | 8.33 (7.99, 8.69) |
| **First month after Index date** |  |  |  |  |  |  |
| Number of subjects | 1,150 | 531 | 619 | 151,926 | 114,229 | 37,697 |
| Mean unadjusted rate of events (events/1000 person-years) | 117.42 | 138.31 | 99.40 | 8.06 | 7.51 | 9.74 |
| Incidence rate (95 % CI) | 65.42 (60.97, 69.87) | 99.70 (90.98, 108.42) | 39.38 (34.44, 44.32) | 7.52 (7.29, 7.75) | 6.44 (6.27, 6.61) | 9.74 (8.99, 10.49) |
| **After first month and onwards** |  |  |  |  |  |  |
| Number of subjects | 260 | 97 | 163 | 99,207 | 81,525 | 17,682 |
| Mean unadjusted rate of events (events/1000 person-years) | 13.68 | Diskr | 22.16 | 4.36 | 3.74 | 8.71 |
| Incidence rate (95 % CI) | 8.85 (6.38, 12.26) | 6.234E-8 (6.079E-8, 6.393E-8) | 14.63 (10.07, 21.26) | 5.25 (5.12, 5.39) | 3.49 (3.40, 3.58) | 7.33 (6.93, 7.75) |
| **Patients >= 65 years** |  |  |  |  |  |  |
| Number of subjects | 929 | 437 | 492 | 94,254 | 73,206 | 21,048 |
| Mean unadjusted rate of events (events/1000 person-years) | 102.99 | 121.45 | 89.41 | 6.86 | 5.72 | 15.36 |
| Incidence rate (95 % CI) | 79.03 (70.97, 88.00) | 112.02 (96.64, 129.83) | 73.82 (62.73, 86.88) | 8.85 (8.63, 9.08) | 5.67 (5.53, 5.82) | 13.49 (12.82, 14.19) |
| **Sensitivity analysis. Incidence of diagnosed hyponatraemia in Germany.** | | | | | | |
| **All patients – All exposure time** |  |  |  |  |  |  |
| Number of subjects | 2,701 | 1,827 | 874 | 473,418 | 320,384 | 153,034 |
| Mean unadjusted rate of events (events/1000 person-years) | 8.36 | 7.65 | 9.60 | 0.80 | 0.63 | 1.55 |
| Incidence rate (95 % CI) | 5.03 (4.72, 5.36) | 4.39 (4.04, 4.77) | 5.28 (4.79, 5.82) | 0.80 (0.78, 0.81) | 0.55 (0.54, 0.55) | 1.07 (1.04, 1.10) |
| **First month after Index date** |  |  |  |  |  |  |
| Number of subjects | 2,701 | 1,827 | 874 | 473,418 | 320,384 | 153,034 |
| Mean unadjusted rate of events (events/1000 person-years) | 31.69 | 20.08 | 55.95 | 2.74 | 2.05 | 4.29 |
| Incidence rate (95 % CI) | 17.88 (17.02, 18.79) | 10.95 (10.24, 11.71) | 28.42 (26.26, 30.77) | 2.40 (2.37, 2.44) | 1.76 (1.73, 1.79) | 3.25 (3.16, 3.34) |
| **After first month and onwards** |  |  |  |  |  |  |
| Number of subjects | 2,411 | 1,551 | 860 | 425,794 | 305,124 | 120,670 |
| Mean unadjusted rate of events (events/1000 person-years) | 4.12 | 5.23 | 2.22 | 0.59 | 0.50 | 1.00 |
| Incidence rate (95 % CI) | 2.29 (2.10, 2.50) | 2.74 (2.48, 3.04) | 1.23 (1.04, 1.46) | 0.56 (0.56, 0.57) | 0.43 (0.43, 0.44) | 0.65 (0.63, 0.67) |
| **Patients >= 65 years** |  |  |  |  |  |  |
| Number of subjects | 2,165 | 1,495 | 670 | 250,717 | 172,675 | 78,042 |
| Mean unadjusted rate of events (events/1000 person-years) | 10.37 | 9.12 | 12.83 | 1.07 | 0.81 | 2.35 |
| Incidence rate (95 % CI) | 7.68 (7.10, 8.30) | 6.25 (5.65, 6.92) | 8.97 (7.90, 10.18) | 1.15 (1.13, 1.17) | 0.73 (0.71, 0.74) | 1.72 (1.65, 1.79) |
| **Incidence rate of all-cause mortality in Sweden.** | | | | | | |
| All patients – All exposure time |  |  |  |  |  |  |
| Number of subjects | 2,894 | 977 | 1,917 | 467,462 | 353,718 | 113,744 |
| Mean unadjusted rate of events (events/1000 person-years) | 31.64 | 54.61 | 19.40 | 33.92 | 36.95 | 19.45 |
| Incidence rate (95 % CI) | 15.19 (12.90, 17.88) | 26.78 (21.36, 33.56) | 9.21 (7.62, 11.13) | 15.33 (15.09, 15.57) | 18.94 (18.64, 19.25) | 13.21 (12.91, 13.51) |
| **First month after Index date** |  |  |  |  |  |  |
| Number of subjects | 2,894 | 977 | 1,917 | 467,462 | 353,718 | 113,744 |
| Mean unadjusted rate of events (events/1000 person-years) | 25.26 | 49.94 | 12.71 | 25.65 | 28.67 | 16.28 |
| Incidence rate (95 % CI) | 11.22 (9.82, 12.82) | 21.77 (18.34, 25.84) | 5.76 (4.76, 6.97) | 12.74 (12.49, 13.00) | 15.38 (15.06, 15.71) | 11.30 (10.97, 11.65) |
| **After first month and onwards** |  |  |  |  |  |  |
| Number of subjects | 2,840 | 960 | 1,880 | 460,521 | 348,474 | 112,047 |
| Mean unadjusted rate of events (events/1000 person-years) | 27.89 | 47.40 | 17.49 | 32.75 | 35.76 | 18.41 |
| Incidence rate (95 % CI) | 13.35 (11.27, 15.82) | 23.15 (18.27, 29.33) | 8.29 (6.84, 10.04) | 14.61 (14.38, 14.85) | 18.25 (17.97, 18.55) | 12.47 (12.19, 12.76) |
| **Patients >= 65 years** |  |  |  |  |  |  |
| Number of subjects | 2,360 | 777 | 1,583 | 309,577 | 245,412 | 64,165 |
| Mean unadjusted rate of events (events/1000 person-years) | 36.41 | 64.42 | 22.19 | 42.52 | 44.87 | 28.37 |
| Incidence rate (95 % CI) | 26.19 (21.47, 31.95) | 46.30 (35.29, 60.74) | 15.87 (12.48, 20.18) | 27.26 (26.83, 27.69) | 33.98 (33.57, 34.39) | 23.34 (22.77, 23.93) |
| **Incidence rate of all-cause mortality in Denmark.** | | | | | | |
| **All patients - All exposure time** |  |  |  |  |  |  |
| Number of subjects | 1,150 | 531 | 619 | 151,926 | 114,229 | 37,697 |
| Mean unadjusted rate of events (events/1000 person-years) | 34.76 | 40.87 | 29.76 | 31.96 | 33.58 | 24.45 |
| Incidence rate (95 % CI) | 20.36 (16.01, 25.90) | 24.16 (17.01, 34.32) | 17.46 (13.25, 23.01) | 21.28 (20.78, 21.79) | 24.43 (23.90, 24.97) | 18.87 (18.18, 19.59) |
| **First month after Index date** |  |  |  |  |  |  |
| Number of subjects | 1,150 | 531 | 619 | 151,926 | 114,229 | 37,697 |
| Mean unadjusted rate of events (events/1000 person-years) | 42.35 | 69.06 | 19.75 | 34.80 | 38.81 | 22.68 |
| Incidence rate (95 % CI) | 25.55 (19.25, 31.85) | 43.84 (30.21, 57.47) | 9.79 (5.59, 13.98) | 25.60 (24.70, 26.50) | 30.43 (29.59, 31.27) | 15.46 (14.53, 16.39) |
| **After first month and onwards** |  |  |  |  |  |  |
| Number of subjects | 1,054 | 442 | 612 | 143,722 | 109,835 | 33,887 |
| Mean unadjusted rate of events (events/1000 person-years) | 26.98 | 28.11 | 26.08 | 29.42 | 30.95 | 22.27 |
| Incidence rate (95 % CI) | 15.80 (12.13, 20.59) | 16.60 (11.01, 25.03) | 15.48 (11.60, 20.67) | 19.43 (18.97, 19.90) | 22.35 (21.86, 22.85) | 17.36 (16.71, 18.04) |
| **Patients >= 65 years** |  |  |  |  |  |  |
| Number of subjects | 929 | 437 | 492 | 94254 | 73206 | 21048 |
| Mean unadjusted rate of events (events/1000 person-years) | 38.86 | 47.53 | 31.48 | 42.11 | 43.31 | 35.68 |
| Incidence rate (95 % CI) | 32.17 (23.97, 43.16) | 40.28 (26.70, 60.77) | 26.33 (18.37, 37.76) | 34.54 (33.63, 35.47) | 39.42 (38.60, 40.26) | 31.36 (30.04, 32.74) |
| **Incidence rate of all-cause mortality in Germany.** | | | | | | |
| **All patients - All exposure time** |  |  |  |  |  |  |
| Number of subjects | 2,701 | 1,827 | 874 | 473,418 | 320,384 | 153,034 |
| Mean unadjusted rate of events (events/1000 person-years) | 21.59 | 22.96 | 19.19 | 44.17 | 45.97 | 36.20 |
| Incidence rate (95 % CI) | 10.61 (8.91, 12.64) | 11.46 (9.06, 14.48) | 10.41 (8.33, 13.02) | 30.36 (29.99, 30.74) | 34.55 (34.15, 34.97) | 26.42 (25.92, 26.93) |
| **First month after Index date** |  |  |  |  |  |  |
| Number of subjects | 2,701 | 1,827 | 874 | 473,418 | 320,384 | 153,034 |
| Mean unadjusted rate of events (events/1000 person-years) | 0.00 | 0.00 | 0.00 | 49.83 | 56.29 | 35.47 |
| Incidence rate (95 % CI) | 0.00 (N/A) | 0.00 (N/A) | 0.00 (N/A) | 34.25 (33.77, 34.75) | 42.81 (42.15, 43.48) | 29.94 (29.30, 30.60) |
| **After first month and onwards** |  |  |  |  |  |  |
| Number of subjects | 2,411 | 1,551 | 860 | 425,794 | 305,124 | 120,670 |
| Mean unadjusted rate of events (events/1000 person-years) | 25.52 | 27.44 | 22.25 | 43.55 | 45.03 | 36.34 |
| Incidence rate (95 % CI) | 12.71 (10.69, 15.12) | 13.82 (11.00, 17.36) | 11.82 (9.42, 14.83) | 29.97 (29.58, 30.37) | 33.86 (33.45, 34.27) | 25.65 (25.10, 26.21) |
| **Patients ≥ 65 years** |  |  |  |  |  |  |
| Number of subjects | 2,165 | 1,495 | 670 | 250,717 | 172,675 | 78,042 |
| Mean unadjusted rate of events (events/1000 person-years) | 25.92 | 26.05 | 25.66 | 64.66 | 66.19 | 57.12 |
| Incidence rate (95 % CI) | 17.69 (14.15, 22.12) | 18.63 (13.80, 25.16) | 18.31 (13.78, 24.32) | 50.50 (49.72, 51.29) | 57.29 (56.51, 58.09) | 44.17 (43.07, 45.29) |
| **Incidence of major cardiovascular events in Sweden.** | | | | | | |
| **All patients – All exposure time** |  |  |  |  |  |  |
| Number of subjects | 2,894 | 977 | 1,917 | 467,462 | 353,718 | 113,744 |
| Mean unadjusted rate of events (events/1000 person-years) | 10.76 | 16.38 | 7.76 | 12.15 | 13.42 | 6.10 |
| Incidence rate (95 % CI) | 8.18 (6.76, 9.90) | 12.67 (9.52, 16.86) | 4.37 (3.64, 5.24) | 7.45 (7.34, 7.57) | 10.57 (10.45, 10.70) | 4.62 (4.52, 4.73) |
| **First month after Index date** |  |  |  |  |  |  |
| Number of subjects | 2,894 | 977 | 1,917 | 467,462 | 353,718 | 113,744 |
| Mean unadjusted rate of events (events/1000 person-years) | 8.42 | 12.47 | 6.35 | 13.58 | 15.98 | 6.10 |
| Incidence rate (95 % CI) | 5.66 (4.73, 6.76) | 8.52 (6.49, 11.18) | 2.93 (2.45, 3.51) | 8.02 (7.86, 8.17) | 12.57 (12.37, 12.78) | 4.26 (4.11, 4.41) |
| **After first month and onwards** |  |  |  |  |  |  |
| Number of subjects | 2,840 | 960 | 1,880 | 460,521 | 348,474 | 112,047 |
| Mean unadjusted rate of events (events/1000 person-years) | 9.51 | 14.58 | 6.80 | 11.53 | 12.75 | 5.70 |
| Incidence rate (95 % CI) | 7.22 (5.92, 8.81) | 11.25 (8.36, 15.13) | 3.86 (3.20, 4.67) | 7.02 (6.91, 7.12) | 10.01 (9.89, 10.14) | 4.35 (4.25, 4.46) |
| **Patients >= 65 years** |  |  |  |  |  |  |
| Number of subjects | 2,360 | 777 | 1,583 | 309,577 | 245,412 | 64,165 |
| Mean unadjusted rate of events (events/1000 person-years) | 13.17 | 20.71 | 9.34 | 14.46 | 15.40 | 8.81 |
| Incidence rate (95 % CI) | 12.38 (9.93, 15.44) | 18.99 (13.74, 26.24) | 8.02 (6.39, 10.06) | 10.95 (10.76, 11.14) | 14.49 (14.32, 14.65) | 8.23 (8.04, 8.44) |
| **Incidence rate of major cardiovascular events in Denmark** | | | | | | |
| **All patients - All exposure time** |  |  |  |  |  |  |
| Number of subjects | 1,150 | 531 | 619 | 151,926 | 114,229 | 37,697 |
| Mean unadjusted rate of events (events/1000 person-years) | 6.13 | 4.54 | 7.44 | 7.38 | 7.99 | 4.59 |
| Incidence rate (95 % CI) | 4.66 (3.43, 6.33) | 3.56 (2.01, 6.31) | 4.91 (3.77, 6.38) | 5.47 (5.33, 5.61) | 7.14 (7.00, 7.28) | 3.86 (3.71, 4.01) |
| **First month after Index date** |  |  |  |  |  |  |
| Number of subjects | 1,150 | 531 | 619 | 151,926 | 114,229 | 37,697 |
| Mean unadjusted rate of events (events/1000 person-years) | 21.18 | 23.02 | 19.75 | 8.20 | 10.05 | 2.59 |
| Incidence rate (95 % CI) | 14.55 (12.22, 16.87) | 17.49 (12.88, 22.10) | 0.00488 (0, Infinity) | 5.07 (4.87, 5.27) | 8.77 (8.56, 8.98) | 0.000907 (0. Infinity) |
| **After first month and onwards** |  |  |  |  |  |  |
| Number of subjects | 1,054 | 442 | 612 | 143,722 | 109,835 | 33,887 |
| Mean unadjusted rate of events (events/1000 person-years) | Diskr | Diskr | Diskr | 6.79 | 7.31 | 4.36 |
| Incidence rate (95 % CI) | 1.60 (0.95, 2.68) | 1.954E-8 (1.915E-8, 1.993E-8) | 2.52 (1.72, 3.68) | 5.11 (4.98, 5.24) | 6.54 (6.41, 6.67) | 3.73 (3.59, 3.88) |
| **Patients >= 65 years** |  |  |  |  |  |  |
| Number of subjects | 929 | 437 | 492 | 94,254 | 73,206 | 21,048 |
| Mean unadjusted rate of events (events/1000 person-years) | 7.29 | 5.28 | 8.99 | 9.07 | 9.56 | 6.44 |
| Incidence rate (95 % CI) | 6.75 (4.74, 9.61) | 5.02 (2.61, 9.64) | 7.85 (5.70, 10.81) | 7.58 (7.36, 7.82) | 9.44 (9.24, 9.65) | 5.87 (5.60, 6.14) |
| **Incidence of major cardiovascular events in Germany.** | | | | | | |
| **All patients - All exposure time** |  |  |  |  |  |  |
| Number of subjects | 2,701 | 1,827 | 874 | 473,418 | 320,384 | 153,034 |
| Mean unadjusted rate of events (events/1000 person-years) | 25.77 | 29.52 | 19.19 | 15.57 | 16.54 | 11.26 |
| Incidence rate (95 % CI) | 16.03 (14.34, 17.92) | 19.30 (16.66, 22.37) | 12.01 (10.41, 13.85) | 11.78 (11.62, 11.94) | 14.36 (14.20, 14.53) | 8.70 (8.53, 8.88) |
| **First month after Index date** |  |  |  |  |  |  |
| Number of subjects | 2,701 | 1,827 | 874 | 473,418 | 320,384 | 153,034 |
| Mean unadjusted rate of events (events/1000 person-years) | 31.69 | 40.16 | 13.99 | 18.24 | 21.27 | 11.51 |
| Incidence rate (95 % CI) | 17.26 (15.49, 19.24) | 26.09 (23.00, 29.61) | 8.15 (6.50, 10.22) | 14.38 (14.17, 14.59) | 20.13 (19.84, 20.42) | 9.69 (9.45, 9.92) |
| **After first month and onwards** |  |  |  |  |  |  |
| Number of subjects | 2,411 | 1,551 | 860 | 425,794 | 305,124 | 120,670 |
| Mean unadjusted rate of events (events/1000 person-years) | 24.70 | 27.44 | 20.02 | 15.27 | 16.10 | 11.21 |
| Incidence rate (95 % CI) | 15.43 (13.66, 17.44) | 17.67 (15.02, 20.78) | 12.67 (10.89, 14.74) | 11.48 (11.31, 11.65) | 13.86 (13.69, 14.02) | 8.51 (8.32, 8.71) |
| **Patients ≥ 65 years** |  |  |  |  |  |  |
| Number of subjects | 2,165 | 1,495 | 670 | 250,717 | 172,675 | 78,042 |
| Mean unadjusted rate of events (events/1000 person-years) | 28.51 | 29.96 | 25.66 | 21.10 | 21.85 | 17.41 |
| Incidence rate (95 % CI) | 22.80 (19.78, 26.27) | 25.03 (20.72, 30.23) | 21.16 (17.65, 25.37) | 17.34 (17.04, 17.65) | 19.93 (19.64, 20.23) | 14.45 (14.07, 14.83) |
| **Incidence of major venous thromboembolic events in Sweden.** | | | | | | |
| **All patients – All exposure time** |  |  |  |  |  |  |
| Number of subjects | 2,894 | 977 | 1,917 | 467,462 | 353,718 | 113,744 |
| Mean unadjusted rate of events (events/1000 person-years) | 13.29 | 14.56 | 12.61 | 13.58 | 13.78 | 12.61 |
| Incidence rate (95 % CI) | 11.42 (9.46, 13.78) | 13.07 (9.49, 17.98) | 9.87 (8.10, 12.04) | 12.23 (12.09, 12.37) | 12.43 (12.29, 12.56) | 11.82 (11.61, 12.03) |
| **First month after Index date** |  |  |  |  |  |  |
| Number of subjects | 2,894 | 977 | 1,917 | 467,462 | 353,718 | 113,744 |
| Mean unadjusted rate of events (events/1000 person-years) | 21.05 | 37.42 | 12.71 | 16.78 | 17.67 | 14.03 |
| Incidence rate (95 % CI) | 18.07 (15.94, 20.49) | 32.92 (27.89, 38.86) | 9.38 (7.81, 11.27) | 15.08 (14.86, 15.29) | 16.60 (16.38, 16.83) | 13.40 (13.08, 13.73) |
| **After first month and onwards** |  |  |  |  |  |  |
| Number of subjects | 2,840 | 960 | 1,880 | 460,521 | 348,474 | 112,047 |
| Mean unadjusted rate of events (events/1000 person-years) | 10.14 | 9.12 | 10.69 | 12.81 | 13.05 | 11.71 |
| Incidence rate (95 % CI) | 8.69 (7.04, 10.72) | 8.13 (5.47, 12.07) | 8.38 (6.80, 10.32) | 11.42 (11.29, 11.56) | 11.68 (11.55, 11.81) | 10.97 (10.78, 11.17) |
| **Patients >= 65 years** |  |  |  |  |  |  |
| Number of subjects | 2,360 | 777 | 1,583 | 309,577 | 245,412 | 64,165 |
| Mean unadjusted rate of events (events/1000 person-years) | 15.49 | 18.40 | 14.01 | 15.27 | 15.11 | 16.18 |
| Incidence rate (95 % CI) | 14.82 (11.93, 18.41) | 18.08 (12.69, 25.76) | 13.24 (10.39, 16.86) | 15.47 (15.26, 15.68) | 14.97 (14.80, 15.14) | 15.91 (15.56, 16.26) |
| **Incidence of major venous thromboembolic events in Denmark.** | | | | | | |
| **All patients - All exposure time** |  |  |  |  |  |  |
| Number of subjects | 1,150 | 531 | 619 | 151,926 | 114,229 | 37,697 |
| Mean unadjusted rate of events (events/1000 person-years) | 10.22 | 4.54 | 14.88 | 10.18 | 10.21 | 10.07 |
| Incidence rate (95 % CI) | 8.63 (6.55, 11.35) | 3.86 (2.04, 7.33) | 12.43 (9.55, 16.18) | 9.67 (9.48, 9.87) | 9.59 (9.41, 9.77) | 9.72 (9.41, 10.03) |
| **First month after Index date** |  |  |  |  |  |  |
| Number of subjects | 1,150 | 531 | 619 | 151,926 | 114,229 | 37,697 |
| Mean unadjusted rate of events (events/1000 person-years) | Diskr | Diskr | Diskr | 13.91 | 14.54 | 11.99 |
| Incidence rate (95 % CI) | 1.756E-8 (1.71E-8, 1.801E-8) | 5.108E-8 (5.032E-8, 5.289E-8) | 6.200E-9 (5.951E-9, 6.453E-9) | 14.44 (14.05, 14.83) | 13.73 (13.73, 14.08) | 11.84 (11.33, 12.35) |
| **After first month and onwards** |  |  |  |  |  |  |
| Number of subjects | 1,054 | 442 | 612 | 143,722 | 109,835 | 33,887 |
| Mean unadjusted rate of events (events/1000 person-years) | 10.38 | 4.68 | 14.90 | 9.16 | 9.22 | 8.89 |
| Incidence rate (95 % CI) | 8.72 (6.68, 11.38) | 3.98 (2.15, 7.38) | 12.26 (9.47, 15.87) | 8.62 (8.44, 8.80) | 8.65 (8.48, 8.81) | 8.51 (8.23, 8.81) |
| **Patients >= 65 years** |  |  |  |  |  |  |
| Number of subjects | 929 | 437 | 492 | 94,254 | 73,206 | 21,048 |
| Mean unadjusted rate of events (events/1000 person-years) | 12.15 | 5.28 | 17.99 | 11.93 | 11.72 | 13.05 |
| Incidence rate (95 % CI) | 11.72 (8.60, 15.97) | 5.12 (2.51, 10.46) | 18.20 (13.41, 24.70) | 12.27 (11.97, 12.58) | 11.70 (11.46, 11.96) | 13.08 (12.57, 13.61) |
| **Incidence rate of major venous thromboembolic events in Germany.** | | | | | | |
| **All patients - All exposure time** |  |  |  |  |  |  |
| Number of subjects | 2,701 | 1,827 | 874 | 473,418 | 320,384 | 153,034 |
| Mean unadjusted rate of events (events/1000 person-years) | 5.57 | 3.28 | 9.60 | 3.66 | 3.46 | 4.54 |
| Incidence rate (95 % CI) | 4.98 (4.36, 5.69) | 2.86 (2.27, 3.60) | 8.07 (6.99, 9.31) | 3.85 (3.80, 3.90) | 3.35 (3.31, 3.39) | 4.37 (4.29, 4.46) |
| **First month after Index date** |  |  |  |  |  |  |
| Number of subjects | 2,701 | 1,827 | 874 | 473,418 | 320,384 | 153,034 |
| Mean unadjusted rate of events (events/1000 person-years) | 9.05 | 0.00 | 27.97 | 5.33 | 5.41 | 5.15 |
| Incidence rate (95 % CI) | 7.51 (6.67, 8.45) | 0.00 (N/A) | 20.52 (18.28, 23.02) | 5.41 (5.34, 5.48) | 5.57 (5.49, 5.65) | 4.94 (4.83, 5.05) |
| **After first month and onwards** |  |  |  |  |  |  |
| Number of subjects | 2,411 | 1,551 | 860 | 425,794 | 305,124 | 120,670 |
| Mean unadjusted rate of events (events/1000 person-years) | 4.94 | 3.92 | 6.67 | 3.48 | 3.29 | 4.42 |
| Incidence rate (95 % CI) | 4.43 (3.82, 5.14) | 3.40 (2.73, 4.23) | 5.76 (4.79, 6.92) | 3.67 (3.62, 3.72) | 3.15 (3.11, 3.20) | 4.26 (4.17, 4.35) |
| **Patients ≥ 65 years** |  |  |  |  |  |  |
| Number of subjects | 2,165 | 1,495 | 670 | 250,717 | 172,675 | 78,042 |
| Mean unadjusted rate of events (events/1000 person-years) | 4.32 | 2.61 | 7.70 | 4.28 | 3.94 | 5.95 |
| Incidence rate (95 % CI) | 4.22 (3.48, 5.12) | 2.44 (1.77, 3.36) | 7.12 (5.72, 8.86) | 4.66 (4.58, 4.74) | 3.83 (3.76, 3.89) | 5.63 (5.48, 5.79) |
| **Incidence of acute exacerbation of CHF in Sweden in a smaller population with chronic cardiac insufficiency** | | | | | | |
| **All patients – All exposure time** |  |  |  |  |  |  |
| Number of subjects | 49 | 21 | 28 | 7,536 | 6,176 | 1,360 |
| Mean unadjusted rate of events (events/1000 person-years) | 279.43 | 277.70 | 281.18 | 675.46 | 694.51 | 537.43 |
| Incidence rate (95 % CI) | 232.89 (95.06, 570.53) | 220.39 (59.90, 810.85) | 275.35 (92.76, 817.38) | 522.32 (488.94, 557.98) | 575.89 (542.25, 611.63) | 539.56 (480.98, 605.27) |
| **First month after Index date** |  |  |  |  |  |  |
| Number of subjects | 49 | 21 | 28 | 7,536 | 6,176 | 1,360 |
| Mean unadjusted rate of events (events/1000 person-years) | 249.32 | 0.00 | 437.43 | 832.18 | 864.15 | 686.81 |
| Incidence rate (95 % CI) | 216.29 (58.96, 793.35) | 1.322E-6 (1.179E-6, 1.483E-6) | 481.39 (139.17, 1665.14) | 679.69 (607.20, 760.83) | 704.41 (628.20, 789.87) | 800.26 (659.74, 970.71) |
| **After first month and onwards** |  |  |  |  |  |  |
| Number of subjects | 47 | 21 | 26 | 7,332 | 6,015 | 1,317 |
| Mean unadjusted rate of events (events/1000 person-years) | 245.70 | 277.70 | 212.97 | 632.67 | 653.02 | 484.88 |
| Incidence rate (95 % CI) | 202.81 (79.68, 516.19) | 219.67 (61.39, 786.09) | 208.32 (62.89, 690.04) | 478.17 (446.87, 511.65) | 539.88 (507.89, 573.90) | 482.96 (429.61, 542.95) |
| **Patients >= 65 years** |  |  |  |  |  |  |
| Number of subjects | 47 | 21 | 26 | 6,982 | 5,697 | 1,285 |
| Mean unadjusted rate of events (events/1000 person-years) | 302.99 | 277.70 | 333.33 | 691.71 | 713.51 | 531.58 |
| Incidence rate (95 % CI) | 278.39 (113.29, 684.13) | 262.28 (70.60, 974.29) | 314.26 (107.05, 922.49) | 590.08 (558.64, 623.29) | 685.34 (660.14, 711.51) | 509.67 (460.25, 564.41) |
| **Incidence of acute exacerbation of heart failure in Germany in subgroup of patients with chronic heart failure.** | | | | | | |
| **All patients - All exposure time** |  |  |  |  |  |  |
| Number of subjects | 390 | 273 | 117 | 39,731 | 27,224 | 12,507 |
| Mean unadjusted rate of events (events/1000 person-years) | 59.57 | 64.44 | 48.55 | 74.59 | 77.87 | 59.46 |
| Incidence rate (95 % CI) | 40.13 (28.24, 57.04) | 46.53 (29.81, 72.63) | 36.35 (21.58, 61.23) | 39.89 (37.92, 41.97) | 45.97 (43.75, 48.29) | 37.06 (33.74, 40.72) |
| **First month after Index date** |  |  |  |  |  |  |
| Number of subjects | 390 | 273 | 117 | 39,731 | 27,224 | 12,507 |
| Mean unadjusted rate of events (events/1000 person-years) | 62.48 | 89.30 | 0.00 | 100.27 | 119.14 | 57.16 |
| Incidence rate (95 % CI) | 33.63 (22.17, 51.03) | 60.06 (38.22, 94.37) | 0.00 (N/A) | 60.53 (57.05, 64.23) | 91.03 (85.84, 96.54) | 43.08 (38.62, 48.04) |
| **After first month and onwards** |  |  |  |  |  |  |
| Number of subjects | 348 | 232 | 116 | 36,065 | 25,936 | 10,129 |
| Mean unadjusted rate of events (events/1000 person-years) | 59.02 | 59.69 | 57.50 | 72.13 | 74.57 | 59.87 |
| Incidence rate (95 % CI) | 40.83 (28.19, 59.13) | 43.77 (27.21, 70.43) | 42.05 (24.86, 71.12) | 37.65 (35.69, 39.73) | 42.17 (40.08, 44.37) | 35.13 (31.51, 39.16) |
| **Patients ≥ 65 years** |  |  |  |  |  |  |
| Number of subjects | 380 | 265 | 115 | 34,782 | 23,285 | 11,497 |
| Mean unadjusted rate of events (events/1000 person-years) | 62.17 | 66.72 | 51.61 | 80.68 | 84.86 | 62.42 |
| Incidence rate (95 % CI) | 52.11 (36.37, 74.67) | 60.76 (38.36, 96.24) | 45.58 (26.97, 77.02) | 50.83 (48.62, 53.14) | 59.12 (56.79, 61.55) | 45.06 (41.75, 48.62) |

Very low number are given in scientific notation, e.g. 1.954E-8 and could be considered as zero, but the Poisson regression is given a low number. N/A = Not applicable.

**Incidence rates of diagnosed hyponatraemia, mortality, MACE and VTE events for patients with and without contraindications in Sweden and Germany. Only ODT patients.**

|  | **ODT patients without contraindications** | **ODT, Male without contraindications** | **ODT, Female without contraindications** | **ODT with contraindications** | **ODT with contraindications, Male** | **ODT with contraindications, Female** |
| --- | --- | --- | --- | --- | --- | --- |
| **Incidence rates of diagnosed hyponatraemia in Sweden** | | | | | | |
| **All patients – All exposure time** |  |  |  |  |  |  |
| Number of subjects | 2,635 | 882 | 1,753 | 259 | 95 | 164 |
| Mean unadjusted rate of events (events/1000 person-years) | 0.00 | 0.00 | 0.00 | 193.53 | 177.82 | 202.03 |
| Incidence rate (95 % CI) | 1.15E-10 (9.9E-11, 1.33E-10) | 1.2E-10 (1.01E-10, 1.43E-10) | 1.8E-14 (0, Infinity) | 165.63 (142.71, 192.22) | 170.71 (143.03, 203.75) | 0.060128 (0, Infinity) |
| **First month after Index date** |  |  |  |  |  |  |
| Number of subjects | 2,635 | 882 | 1,753 | 259 | 95 | 164 |
| Mean unadjusted rate of events (events/1000 person-years) | 0.00 | 0.00 | 0.00 | 334.50 | 391.76 | 301.45 |
| Incidence rate (95 % CI) | 9.87E-14 (0, Infinity) | 7.93E-14 (0, Infinity) | 1.13E-13 (0, Infinity) | 0.127934 (0, Infinity) | 0.116807 (0, Infinity) | 0.147651 (0, Infinity) |
| **After first month and onwards** |  |  |  |  |  |  |
| Number of subjects | 2,581 | 865 | 1,716 | 259 | 95 | 164 |
| Mean unadjusted rate of events (events/1000 person-years) | 0.00 | 0.00 | 0.00 | 150.33 | 125.02 | 164.00 |
| Incidence rate (95 % CI) | 4.16E-11 (3.65E-11, 4.74E-11) | 4.31E-11 (3.69E-11, 5.04E-11) | 1.83E-14 (0, Infinity) | 138.15 (121.14, 157.55) | 126.19 (107.94, 147.52) | 0.050722 (0, Infinity) |
| Standard Error of Mean | 0.00 | 0.00 | 0.00 | 9.26 | 10.06 | 845.48 |
| **Patients >= 65 years** |  |  |  |  |  |  |
| Number of subjects | 2,113 | 689 | 1,424 | 247 | 88 | 159 |
| Mean unadjusted rate of events (events/1000 person-years) | 0.00 | 0.00 | 0.00 | 199.32 | 182.17 | 207.70 |
| Incidence rate (95 % CI) | 1.28E-10 (1.17E-10, 1.41E-10) | 1.24E-10 (1.06E-10, 1.46E-10) | 1.34E-10 (1.19E-10, 1.52E-10) | 205.06 (186.50, 225.47) | 184.35 (156.58, 217.04) | 213.84 (189.32, 241.53) |
| **Incidence rates of diagnosed hyponatraemia in Germany** | | | | | | |
| **All patients – All exposure time** |  |  |  |  |  |  |
| Number of subjects | 2,096 | 1,420 | 676 | 605 | 407 | 198 |
| Mean unadjusted rate of events (events/1000 person-years) | 11.56 | 11.19 | 12.21 | 25.72 | 15.03 | 44.86 |
| Incidence rate (95 % CI) | 0.00 (N/A) | 0.00 (N/A) | 0.00 (N/A) | 0.01 (N/A) | 0.00 (N/A) | 0.00 (N/A) |
| **First month after Index date** |  |  |  |  |  |  |
| Number of subjects | 2,096 | 1,420 | 676 | 605 | 407 | 198 |
| Mean unadjusted rate of events (events/1000 person-years) | 17.51 | 17.22 | 18.10 | 80.77 | 30.05 | 184.69 |
| Incidence rate (95 % CI) | 0.01 (N/A) | 0.01 (N/A) | 0.00 (N/A) | 0.03 (N/A) | 0.01 (N/A) | 0.00 (N/A) |
| **After first month and onwards** |  |  |  |  |  |  |
| Number of subjects | 1,871 | 1,206 | 665 | 540 | 345 | 195 |
| Mean unadjusted rate of events (events/1000 person-years) | 10.49 | 10.02 | 11.29 | 15.29 | 12.02 | 21.00 |
| Incidence rate (95 % CI) | 0.00 (N/A) | 0.00 (N/A) | 0.00 (N/A) | 0.00 (N/A) | 0.00 (N/A) | 0.00 (N/A) |
| **Patients >= 65 years** |  |  |  |  |  |  |
| Number of subjects | 1,592 | 1,106 | 486 | 573 | 389 | 184 |
| Mean unadjusted rate of events (events/1000 person-years) | 15.01 | 13.85 | 17.34 | 27.44 | 15.78 | 49.31 |
| Incidence rate (95 % CI) | 6.98 ( 4.97, 9.81) | 8.01 ( 5.92, 10.84) | 0.00 (N/A) | 14.07 ( 9.81, 20.18) | 10.62 ( 7.27, 15.50) | 0.00 (N/A) |
| **Incidence of all-cause mortality events with and without contraindications. Sweden.** | | | | | | |
| **All patients – All exposure time** |  |  |  |  |  |  |
| Number of subjects | 2,635 | 882 | 1,753 | 259 | 95 | 164 |
| Mean unadjusted rate of events (events/1000 person-years) | 26.06 | 44.61 | 16.18 | 81.16 | 142.26 | 48.10 |
| Incidence rate (95 % CI) | 14.44 (11.85, 17.59) | 25.91 (19.18, 35.01) | 7.92 (5.92, 10.59) | 36.89 (28.21, 48.24) | 73.99 (48.99, 111.74) | 17.09 (11.53, 25.33) |
| **First month after Index date** |  |  |  |  |  |  |
| Number of subjects | 2,635 | 882 | 1,753 | 259 | 95 | 164 |
| Mean unadjusted rate of events (events/1000 person-years) | 27.75 | 55.33 | 13.89 | 0.00 | 0.00 | 0.00 |
| Incidence rate (95 % CI) | 23.18 (19.61, 27.40) | 54.79 (43.51, 69.00) | 1.075E-6 (0, Infinity) | 3.228E-9 (0, Infinity) | 4.259E-9 (0, Infinity) | 1.11E-17 (0, Infinity) |
| **After first month and onwards** |  |  |  |  |  |  |
| Number of subjects | 2,581 | 865 | 1,716 | 259 | 95 | 164 |
| Mean unadjusted rate of events (events/1000 person-years) | 21.87 | 36.56 | 14.05 | 81.16 | 142.26 | 48.10 |
| Incidence rate (95 % CI) | 10.78 (8.69, 13.36) | 16.74 (11.74, 23.88) | 7.45 (5.65, 9.82) | 32.27 (24.64, 42.27) | 57.18 (37.18, 87.95) | 18.77 (12.93, 27.24) |
| **Patients >= 65 years** |  |  |  |  |  |  |
| Number of subjects | 2,113 | 689 | 1,424 | 247 | 88 | 159 |
| Mean unadjusted rate of events (events/1000 person-years) | 29.81 | 51.91 | 18.54 | 86.37 | 161.93 | 49.45 |
| Incidence rate (95 % CI) | 17.93 (14.48, 22.21) | 32.80 (23.78, 45.23) | 9.38 (6.70, 13.12) | 47.49 (35.98, 62.67) | 98.87 (64.77, 150.92) | 20.43 (13.36, 31.25) |
| **Incidence of all-cause mortality for patients with and without contraindications in Germany** | | | | | | |
| **All patients – All exposure time** |  |  |  |  |  |  |
| Number of subjects | 2,096 | 1,420 | 676 | 605 | 407 | 198 |
| Mean unadjusted rate of events (events/1000 person-years) | 16.90 | 20.98 | 9.77 | 38.57 | 30.05 | 53.83 |
| Incidence rate (95 % CI) | 9.23 ( 7.23, 11.78) | 13.48 (10.41, 17.44) | 0.00 (N/A) | 18.23 (13.76, 24.14) | 17.41 (12.36, 24.52) | 0.01 (N/A) |
| **First month after Index date** |  |  |  |  |  |  |
| Number of subjects | 2,096 | 1,420 | 676 | 605 | 407 | 198 |
| Mean unadjusted rate of events (events/1000 person-years) | 0.00 | 0.00 | 0.00 | 0.00 | 0.00 | 0.00 |
| Incidence rate (95 % CI) | N/A | N/A | N/A | N/A | N/A | N/A |
| **After first month and onwards** |  |  |  |  |  |  |
| Number of subjects | 1,871 | 1,206 | 665 | 540 | 345 | 195 |
| Mean unadjusted rate of events (events/1000 person-years) | 19.93 | 25.05 | 11.29 | 45.88 | 36.07 | 63.01 |
| Incidence rate (95 % CI) | 10.75 ( 8.26, 14.00) | 16.07 (12.06, 21.41) | 0.00 (N/A) | 21.89 (16.20, 29.59) | 21.33 (14.60, 31.15) | 0.02 (N/A) |
| **Patients >= 65 years** |  |  |  |  |  |  |
| Number of subjects | 1,592 | 1,106 | 486 | 573 | 389 | 184 |
| Mean unadjusted rate of events (events/1000 person-years) | 20.79 | 24.24 | 13.87 | 41.17 | 31.56 | 59.18 |
| Incidence rate (95 % CI) | 15.38 (12.46, 18.98) | 19.03 (15.05, 24.06) | 8.93 ( 5.87, 13.59) | 30.91 (24.42, 39.11) | 25.07 (18.35, 34.24) | 38.89 (26.80, 56.43) |
| **Incidence of major cardiovascular events with and without contraindications. Sweden** | | | | | | |
| **All patients – All exposure time** |  |  |  |  |  |  |
| Number of subjects | 2,635 | 882 | 1,753 | 259 | 95 | 164 |
| Mean unadjusted rate of events (events/1000 person-years) | 9.86 | 14.20 | 7.55 | 18.73 | 35.56 | 9.62 |
| Incidence rate (95 % CI) | 0.003554 (0, Infinity) | 0.006313 (0, Infinity) | 0.002502 (0, Infinity) | 0.005493 (0, Infinity) | 0.013925 (0, Infinity) | 0.002284 (0, Infinity) |
| **First month after Index date** |  |  |  |  |  |  |
| Number of subjects | 2,635 | 882 | 1,753 | 259 | 95 | 164 |
| Mean unadjusted rate of events (events/1000 person-years) | 9.25 | 13.81 | 6.95 | 0.00 | 0.00 | 0.00 |
| Incidence rate (95 % CI) | 1.023E-6 (0, Infinity) | 1.164E-6 (0, Infinity) | 8.534E-7 (0, Infinity) | 1.82E-17 (0, Infinity) | 1.65E-17 (0, Infinity) | 1.77E-17 (0, Infinity) |
| **After first month and onwards** |  |  |  |  |  |  |
| Number of subjects | 2,581 | 865 | 1,716 | 259 | 95 | 164 |
| Mean unadjusted rate of events (events/1000 person-years) | 8.47 | 12.19 | 6.49 | 18.73 | 35.56 | 9.62 |
| Incidence rate (95 % CI) | 0.00325 (0, Infinity) | 0.004074 (0, Infinity) | 0.002325 (0, Infinity) | 0.005926 (0, Infinity) | 0.010514 (0, Infinity) | 0.002505 (0, Infinity) |
| **Patients >= 65 years** |  |  |  |  |  |  |
| Number of subjects | 2,113 | 689 | 1,424 | 247 | 88 | 159 |
| Mean unadjusted rate of events (events/1000 person-years) | 12.28 | 18.17 | 9.27 | 19.93 | 40.48 | 9.89 |
| Incidence rate (95 % CI) | 11.40 (9.72, 13.38) | 17.36 (13.56, 22.22) | 6.62 (5.14, 8.52) | 17.63 (12.82, 24.22) | 38.29 (24.41, 60.05) | 6.04 (3.61, 10.11) |
| **Incidence of MACE in Germany for patients with and without contraindications** | | | | | | |
| **All patients – All exposure time** |  |  |  |  |  |  |
| Number of subjects | 2,096 | 1,420 | 676 | 605 | 407 | 198 |
| Mean unadjusted rate of events (events/1000 person-years) | 19.56 | 22.38 | 14.65 | 48.22 | 55.10 | 35.89 |
| Incidence rate (95 % CI) | 12.28 (10.04, 15.02) | 16.14 (12.89, 20.20) | 0.00 (N/A) | 31.28 (24.71, 39.59) | 46.34 (35.52, 60.47) | 0.01 (N/A) |
| **First month after Index date** |  |  |  |  |  |  |
| Number of subjects | 2,096 | 1,420 | 676 | 605 | 407 | 198 |
| Mean unadjusted rate of events (events/1000 person-years) | 23.34 | 25.84 | 18.10 | 60.57 | 90.14 | 0.00 |
| Incidence rate (95 % CI) | 0.01 (N/A) | 0.01 (N/A) | 0.00 (N/A) | 0.02 (N/A) | 0.05 (N/A) | 0.00 (N/A) |
| **After first month and onwards** |  |  |  |  |  |  |
| Number of subjects | 1,871 | 1,206 | 665 | 540 | 345 | 195 |
| Mean unadjusted rate of events (events/1000 person-years) | 18.88 | 21.71 | 14.11 | 45.88 | 48.09 | 42.01 |
| Incidence rate (95 % CI) | 9.82 ( 7.64, 12.63) | 13.05 ( 9.94, 17.14) | 0.00 (N/A) | 24.16 (18.24, 32.00) | 32.68 (23.77, 44.93) | 0.01 (N/A) |
| **Patients >= 65 years** |  |  |  |  |  |  |
| Number of subjects | 1,592 | 1,106 | 486 | 573 | 389 | 184 |
| Mean unadjusted rate of events (events/1000 person-years) | 20.79 | 20.78 | 20.81 | 51.46 | 57.86 | 39.45 |
| Incidence rate (95 % CI) | 14.05 (11.19, 17.65) | 14.50 (11.11, 18.93) | 13.73 ( 9.09, 20.75) | 37.89 (29.97, 47.89) | 45.61 (34.98, 59.48) | 26.28 (16.65, 41.50) |
| **Incidence of VTE with and without contraindications. Sweden.** | | | | | | |
| **All patients – All exposure time** |  |  |  |  |  |  |
| Number of subjects | 2,635 | 882 | 1,753 | 259 | 95 | 164 |
| Mean unadjusted rate of events (events/1000 person-years) | 12.68 | 14.20 | 11.87 | 18.73 | 17.78 | 19.24 |
| Incidence rate (95 % CI) | 9.10 (7.47, 11.07) | 0.004685 (0, Infinity) | 9.56 (7.77, 11.75) | 11.57 (8.15, 16.43) | 0.005189 (0, Infinity) | 13.27 (8.82, 19.98) |
| **First month after Index date** |  |  |  |  |  |  |
| Number of subjects | 2,635 | 882 | 1,753 | 259 | 95 | 164 |
| Mean unadjusted rate of events (events/1000 person-years) | 18.49 | 27.62 | 13.90 | 47.12 | 129.02 | 0.00 |
| Incidence rate (95 % CI) | 0.009728 (0, Infinity) | 0.011736 (0, Infinity) | 0.005735 (0, Infinity) | 0.022288 (0, Infinity) | 0.04763 (0, Infinity) | 2.31E-13 (0, Infinity) |
| **After first month and onwards** |  |  |  |  |  |  |
| Number of subjects | 2,581 | 865 | 1,716 | 259 | 95 | 164 |
| Mean unadjusted rate of events (events/1000 person-years) | 9.88 | 10.16 | 9.73 | 12.49 | 0.00 | 19.24 |
| Incidence rate (95 % CI) | 6.78 (5.62, 8.19) | 0.003346 (0, Infinity) | 7.88 (6.44, 9.65) | 7.08 (4.89, 10.25) | 9.6E-14 (0, Infinity) | 13.13 (8.97, 19.22) |
| **Patients >= 65 years** |  |  |  |  |  |  |
| Number of subjects | 2,113 | 689 | 1,424 | 247 | 88 | 159 |
| Mean unadjusted rate of events (events/1000 person-years) | 14.91 | 18.17 | 13.24 | 19.93 | 20.24 | 19.78 |
| Incidence rate (95 % CI) | 14.79 (12.78, 17.13) | 17.79 (14.05, 22.51) | 12.36 (10.24, 14.92) | 19.18 (13.73, 26.78) | 19.70 (10.70, 36.27) | 17.40 (11.64, 26.02) |
| **Incidence of VTE in Germany for patients with and without contraindications** | | | | | | |
| **All patients – All exposure time** |  |  |  |  |  |  |
| Number of subjects | 2,096 | 1,420 | 676 | 605 | 407 | 198 |
| Mean unadjusted rate of events (events/1000 person-years) | 6.22 | 4.20 | 9.77 | 3.21 | 0.00 | 8.97 |
| Incidence rate (95 % CI) | 6.70 ( 5.77, 7.78) | 4.78 ( 4.07, 5.60) | 9.71 ( 7.34, 12.85) | 4.03 ( 2.75, 5.91) | 0.00 (N/A) | 10.57 ( 6.18, 18.08) |
| **First month after Index date** |  |  |  |  |  |  |
| Number of subjects | 2,096 | 1,420 | 676 | 605 | 407 | 198 |
| Mean unadjusted rate of events (events/1000 person-years) | 11.67 | 0.00 | 36.20 | 0.00 | 0.00 | 0.00 |
| Incidence rate (95 % CI) | 0.00 (N/A) | N/A | 0.01 (N/A) | 0.00 (N/A) | N/A | 0.00 (N/A) |
| **After first month and onwards** |  |  |  |  |  |  |
| Number of subjects | 1,871 | 1,206 | 665 | 540 | 345 | 195 |
| Mean unadjusted rate of events (events/1000 person-years) | 5.25 | 5.01 | 5.65 | 3.82 | 0.00 | 10.50 |
| Incidence rate (95 % CI) | 5.15 ( 4.33, 6.14) | 5.76 ( 4.84, 6.85) | 0.00 (N/A) | 5.29 ( 3.73, 7.49) | 0.00 (N/A) | 0.01 (N/A) |
| **Patients >= 65 years** |  |  |  |  |  |  |
| Number of subjects | 1,592 | 1,106 | 486 | 573 | 389 | 184 |
| Mean unadjusted rate of events (events/1000 person-years) | 4.62 | 3.46 | 6.94 | 3.43 | 0.00 | 9.86 |
| Incidence rate (95 % CI) | 5.22 ( 4.36, 6.24) | 3.87 ( 3.25, 4.61) | 7.17 ( 4.98, 10.34) | 3.75 ( 2.64, 5.33) | 0.00 (N/A) | 10.14 ( 6.13, 16.77) |

Very low number are given in scientific notation, e.g. 1.954E-8 and could be considered as zero, but the Poisson regression is given a low number. N/A = Not applicable.

**Incidence rates (events/1000 patient-years) with 95% CI of CHF exacerbation in a sub-population with previous history of CHF.**

| **Population** | **Germany** | **Sweden** |
| --- | --- | --- |
| CHF - ODT | n=390; rate=40.13 (28.24, 57.04) | n=49, rate=232.92 (95.14, 570.48) |
| CHF - LUTS | n=39,731; rate=39.89 (37.92, 41.97) | n=7,536; rate=522.31 (488.91, 558.03) |

There were 1,150 LUTS and 16 ODT patients in Denmark, too few patients to publish the incidence rates due to GDPR regulations.
